# Supplementary material for: Is there a difference in the analgesic response to intra-articular bupivacaine injection in people with knee osteoarthritis pain with or without central sensitisation? Protocol of a feasibility randomised controlled trial
Source: BMJ Open. 2023 Jul 11;13(7):e072138. doi: 10.1136/bmjopen-2023-072138 (PMC10347485; doi:10.1136/bmjopen-2023-072138)
Supplement: Supplementary data [file bmjopen-2023-072138supp002.pdf]

Nottingham University Hospitals NHS
NHS Trust

# Participant Consent Form

Version: 1.1 – Date: 04/01/2021 – IRAS number: 270642

## Understanding Pain Mechanisms in Knee OA — UP- KNEE study

**Full Title:** Is there a difference in the analgesic response to intra-articular bupivacaine injection in people with knee osteoarthritis pain with or without central sensitisation?: a feasibility randomised controlled trial

**Chief Investigator:** Professor Brigitte Scammell (Nottingham University Hospitals NHS Trust)
**Principal Investigator:** Dr Yasmine Zedan

Participant Study ID: ……………………..                Initials: ………………

**Participant initial each box**

1. I confirm that I have read and understand the information sheet dated ________ (version _____) and the additional information leaflet dated ________ (version _____) for the above study and have had the opportunity to ask questions. ☐
2. I understand that my participation is voluntary and that I am free to withdraw at any time without my medical care or legal rights being affected. ☐
3. I understand that my medical records may be looked at by authorised individuals from the Sponsor for the study, the research group and the UK Regulatory Authority in order to check that the study is being carried out correctly. ☐
4. I understand that even if I withdraw from the above study, the data collected from me will be used in analysing the results of the trial. ☐
5. I consent to the storage including electronic, of personal information for the purposes of this study. I understand that any information that could identify me will be kept strictly confidential and that no personal information will be included in the study report or other publication. ☐
6. I understand that my participation in the study will involve having an injection of either a local anaesthetic (bupivacaine) or placebo (a mixture of salt and water) into my knee joint. ☐
7. I understand that participation in the study will involve MRI scans of my brain and knee and an x-ray of my knee (if needed). ☐
8. I agree that my GP, or any other doctor treating me, will be notified of my participation in this study. ☐
9. I understand that should there be any abnormal findings on either the fMRI scans, knee MRI or x-rays taken as part of this study, I agree to my GP being contacted. ☐
10. I agree to take part in the study. ☐
11. I agree to being contacted regarding future research studies (**OPTIONAL**) YES ☐ NO ☐

| ______________________ | ____________ | ______________________ |
|---|---|---|
| Name of the patient *(Print)* | date | Patient's signature |
| ______________________ | ____________ | ______________________ |
| Name of person receiving consent *(Print)* | date | Signature |

**Original to be retained and filed in the site file. 1 copy to patient, 1 copy to be filed in patient's notes.**

IRAS 270642 UP-KNEE Informed Consent Form Version 1.1, 04JAN2021
