# Supplementary material for: Is there a difference in the analgesic response to intra-articular bupivacaine injection in people with knee osteoarthritis pain with or without central sensitisation? Protocol of a feasibility randomised controlled trial
Source: BMJ Open. 2023 Jul 11;13(7):e072138. doi: 10.1136/bmjopen-2023-072138 (PMC10347485; doi:10.1136/bmjopen-2023-072138)
Supplement: Supplementary data [file bmjopen-2023-072138supp003.pdf]

Supplementary C

Is there a difference in the analgesic response to intra-articular bupivacaine injection in people with knee osteoarthritis pain with or without central sensitisation?: a feasibility randomised controlled trial

Template for intervention description and replication (TIDieR) checklist[1]

1. **BRIEF NAME:**
   Intra-articular knee injection with bupivacaine/placebo
2. **WHY:**
   There is a need for a better mechanistic understanding of individual factors affecting pain relief, consequently informing personalised treatment guidelines. The aim of this research is to conduct a feasibility study in order to provide a strategy for a future randomised clinical trial by comparing the effect of a peripherally targeted intervention (intra-articular bupivacaine injection) versus placebo in pre-surgical knee OA with and without signs of centrally mediated pain mechanisms. We have selected intra-articular injection of bupivacaine to achieve analgesia because this was shown to reduce knee pain in participants with knee OA one hour after injection.[2,3]
3. **WHAT**:
   Materials:
   Bupivacaine is used in clinical practice (combined with corticosteroids) for pain relief. This study does not have the intention to treat. This is not a clinical trial of an IMP.
   Description of bupivacaine (active drug):
   Bupivacaine is a local anaesthetic agent with a slow onset of action of approximately 2-5 minutes after injection and its effects last longer between approximately 2-4 hours after wash out for single knee injection. Bupivacaine binds to the intracellular portion of sodium channels and blocks sodium influx into nerve cells, preventing depolarization and the generation and conduction of nerve impulses. Its chemical designation is 1-butyl-N-(2,6-dimethylphenyl) piperidine-2-carboxamide. The empirical formula is C18H28N20, which corresponds to a molecular mass of 288.43 g/mol. Its pharmaceutical form is in a white crystalline powder that is freely soluble in 95 per cent ethanol, soluble in water, and slightly soluble in chloroform or acetone.

   The substance for placebo is sodium chloride (5 ml, 9mg/ml, 0.9% solution for injection).

   Procedures: Bupivacaine/sodium chloride will be administered in a setting fully equipped for the monitoring and support of respiratory and cardiovascular function. For intra-articular injection, the dosage of bupivacaine (5 ml, 0.25%w/v bupivacaine) and sodium chloride (5 ml, 0.9% solution for injection) will be the same for each participant.
4. **WHO PROVIDED:**
   Bupivacaine/sodium chloride will be administered by personnel experienced in their use, with training from the Nottingham University Hospitals NHS Trust, and have also been trained in advanced life support and anaphylaxis management.

5. **HOW:**
   Bupivacaine/sodium chloride will be administered by the intra-articular route.
6. **WHERE:**
   Bupivacaine/sodium chloride will be administered in a setting fully equipped for the monitoring and support of respiratory and cardiovascular function under totally aseptic conditions.
7. **WHEN and HOW MUCH:**
   The study involves a single visit which will include an assessment to check patients' suitability to undergo intervention with bupivacaine/placebo intra-articular knee injection. During the visit, all participants will be invited to undertake the following assessments: 1) a suite of psychometric questionnaires; 2) Quantitative Sensory Testing; 3) MRI scan of the knee and brain; 4) a 6-Minute Walk Test; and 5) a single intra-articular injection of either bupivacaine (5 ml, 0.25%w/v bupivacaine) or sodium chloride (5 ml, 0.9% solution for injection) into the index knee. Assessments will be repeated post intra-articular injection apart from the knee MRI scan.
8. **TAILORING:**
   N/A
9. **HOW WELL:**
   Planned:
   All clinicians who will deliver the intra-articular injection in this study have been trained in Nottingham University Hospitals NHS Trust, and have also been trained in advanced life support and anaphylaxis management. The clinicians will adhere to a standardised protocol of injection. Protocol completion rates will be collected as one of the study outcomes.

References

1. Hoffmann TC, Glasziou PP, Boutron I, et al. Better reporting of interventions: template for intervention description and replication (TIDieR) checklist and guide. *BMJ*. 2014;348(mar07 3):g1687-g1687. doi:10.1136/bmj.g1687
2. Hassan BS, Doherty SA, Mockett S, Doherty M. Effect of pain reduction on postural sway, proprioception, and quadriceps strength in subjects with knee osteoarthritis. *Ann Rheum Dis*. 2002;61(5):422-428. doi:10.1136/ard.61.5.422
3. Creamer P, Hunt M, Dieppe P. Pain mechanisms in osteoarthritis of the knee: effect of intraarticular anesthetic. *J Rheumatol*. 1996;23(6):1031-1036.
