# Supplementary material for: Is there a difference in the analgesic response to intra-articular bupivacaine injection in people with knee osteoarthritis pain with or without central sensitisation? Protocol of a feasibility randomised controlled trial
Source: BMJ Open. 2023 Jul 11;13(7):e072138. doi: 10.1136/bmjopen-2023-072138 (PMC10347485; doi:10.1136/bmjopen-2023-072138)
Supplement: Supplementary data [file bmjopen-2023-072138supp004.pdf]

Supplementary D:

Quantitative Sensory Testing (QST) protocol:
This protocol follows standardised methodology described in previous studies.[1–5]

Pressure Algometry
To examine pressure pain thresholds (PPT), a handheld pressure algometer (Somedic AB, Sösdala, Sweden) will be used. Pressure will be increased by 30kPa/s until the participant perceives a change of the stimulus from a pressure sensation to pain sensation. The participant will then press a response button and the algometer probe will be immediately withdrawn. The PPT score in kPa is the pressure applied at the time the response button was pressed.
PPT will be recorded from the following sites:
1. Medial joint line of the index knee (the most painful knee): 2 cm proximal to superomedial patellar margin.
2. Lateral joint line of the index knee: 2 cm proximal to superolateral patellar margin.
3. The tibialis anterior muscle as a distant site to examine spreading sensitization.
2. the extensor carpi radialis longus (ECRL) muscle as a remote site to examine widespread hyperalgesia.
A practise test will be done on each site then PPTs will be recorded in triplicates which will then be averaged per site for further analysis.

Cuff Pressure Algometry
A cuff algometer (NociTech and Aalborg University, Denmark) comprised of two 13 cm single chamber tourniquet cuffs (VBM, Sulz, Germany) will use computer-controlled cuff pressure stimuli to assess pain sensitivity of the deep tissue. The device is connected to an air compressor and an electronic VAS rating system (Aalborg University) which is sampled at 10 Hz.
the cuff will be applied at the level of the ipsilateral gastrocnemius muscle and will be inflated at a rate of 1kPa/s until max pressure limit of 100 kPa. The participant will rate his pressure-induced pain severity continuously using the VAS rating system and will press a button to release the pressure. The cuff pain detection threshold (PDT) is the pressure value when the subject rated pain as 1 cm on the VAS rating system.
The cuff pain tolerance threshold (PTT) is the value of maximum pressure at the point the subject had to press the release button because of intolerable pain severity.

Temporal Summation of Pain (TSP) by Cuff Algometry
Ten consecutive cuff pressure stimulations comprised of 1-second stimulus with a 2-second inter-stimulus-interval with an intensity equal to the PTT score will be applied ipsilateral to the index knee. Participants will rate the pain severity continuously throughout the stimulation using the VAS slider and to not return the VAS slider to zero in between cuff stimulations. A constant pressure of 5 kPa is retained by the cuff in-between stimulations to ensure that fixed position of the cuff on the leg during the examination. The VAS score is recorded immediately after each individual cuff stimulus.
TSP score is calculated by subtracting the mean VAS score of the first to 4th cuff stimulations (VAS-I) from the mean VAS score of the 8th to 10th cuff stimulations (VAS-II).

Conditioning Pain Modulation by Cuff Algometry

A painful conditioning stimulus will be applied contralateral to the index knee using the cuff with an inflation pressure equals 70% of the participant's cuff PTT. As a test stimulus, PDT will be simultaneously examined on the ipsilateral side using the second cuff. Conditioning pain modulation (CPM) will be calculated by subtracting the unconditioned PDT score from the conditioned PDT score.

References

1. Petersen KK, Arendt-Nielsen L, Finocchietti S, et al. Age Interactions on Pain Sensitization in Patients With Severe Knee Osteoarthritis and Controls. *Clin J Pain*. 2017;33(12):1081-1087. doi:10.1097/AJP.0000000000000495
2. Rathleff MS, Petersen KK, Arendt-Nielsen L, Thorborg K, Graven-Nielsen T. Impaired Conditioned Pain Modulation in Young Female Adults with Long-Standing Patellofemoral Pain: A Single Blinded Cross-Sectional Study. *Pain Med*. 2016;17(5):980-988. doi:10.1093/pm/pnv017
3. Vaegter HB, Graven-Nielsen T. Pain modulatory phenotypes differentiate subgroups with different clinical and experimental pain sensitivity. *Pain*. 2016;157(7):1480-1488. doi:10.1097/j.pain.0000000000000543
4. Kurien T, Arendt-Nielsen L, Petersen KK, Graven-Nielsen T, Scammell BE. Preoperative Neuropathic Pain-like Symptoms and Central Pain Mechanisms in Knee Osteoarthritis Predicts Poor Outcome 6 Months After Total Knee Replacement Surgery. *J Pain*. 2018;19(11):1329-1341. doi:https://doi.org/10.1016/j.jpain.2018.05.011
5. Iwabuchi SJ, Xing Y, Cottam WJ, et al. Brain perfusion patterns are altered in chronic knee pain: a spatial covariance analysis of arterial spin labelling MRI. *Pain*. 2020;161(6):1255-1263. doi:10.1097/j.pain.0000000000001829
