# Supplementary material for: Is there a difference in the analgesic response to intra-articular bupivacaine injection in people with knee osteoarthritis pain with or without central sensitisation? Protocol of a feasibility randomised controlled trial
Source: BMJ Open. 2023 Jul 11;13(7):e072138. doi: 10.1136/bmjopen-2023-072138 (PMC10347485; doi:10.1136/bmjopen-2023-072138)
Supplement: Author's manuscript [file bmjopen-2023-072138.draft_revisions.pdf]

BMJ Open is committed to open peer review. As part of this commitment we make the peer review history of every article we publish publicly available.

When an article is published we post the peer reviewers' comments and the authors' responses online. We also post the versions of the paper that were used during peer review. These are the versions that the peer review comments apply to.

The versions of the paper that follow are the versions that were submitted during the peer review process. They are not the versions of record or the final published versions. They should not be cited or distributed as the published version of this manuscript.

BMJ Open is an open access journal and the full, final, typeset and author-corrected version of record of the manuscript is available on our site with no access controls, subscription charges or pay-per-view fees (http://bmjopen.bmj.com).

If you have any questions on BMJ Open's open peer review process please email info.bmjopen@bmj.com

BMJ Open

Is there a difference in the analgesic response to intra-articular bupivacaine injection in people with knee osteoarthritis pain with or without central sensitisation?: protocol of a feasibility randomised controlled trial

Journal:	*BMJ Open*
Manuscript ID	bmjopen-2023-072138
Article Type:	Protocol
Date Submitted by the Author:	24-Jan-2023

Complete List of Authors:	Zedan, Yasmine; University of Nottingham, Radiological Sciences, Mental Health & Clinical Neurosciences, School of Medicine; University of Nottingham, Pain Centre Versus Arthritis, School of Medicine<br>Knaggs, Roger; University of Nottingham, Clinical Pharmacy Practice, School of Pharmacy<br>Cooper, Dale; Keele University, School of Allied Health Professions<br>Kurien, Thomas ; University of Nottingham, Pain Centre Versus Arthritis, School of Medicine; University of Nottingham, Academic Orthopaedics, Trauma and Sports Medicine, School of Medicine<br>Walsh, David; University of Nottingham, Pain Centre Versus Arthritis, School of Medicine; University of Nottingham, Academic Rheumatology, School of Medicine<br>Auer, Dorothee; University of Nottingham, Radiological Sciences, Mental Health & Clinical Neurosciences, School of Medicine; University of Nottingham, Pain Centre Versus Arthritis, School of Medicine<br>Scammell, Brigitte; University of Nottingham, Pain Centre Versus Arthritis, School of Medicine; University of Nottingham, Academic Orthopaedics, Trauma and Sports Medicine, School of Medicine

Keywords:	Knee < ORTHOPAEDIC & TRAUMA SURGERY, Musculoskeletal disorders < ORTHOPAEDIC & TRAUMA SURGERY, Protocols & guidelines < HEALTH SERVICES ADMINISTRATION & MANAGEMENT, Magnetic resonance imaging < RADIOLOGY & IMAGING

SCHOLARONE™
Manuscripts

Is there a difference in the analgesic response to intra-articular bupivacaine injection in people with knee osteoarthritis pain with or without central sensitisation?: protocol of a feasibility randomised controlled trial

For peer review only

ABSTRACT

Introduction

Pain is the main symptom of osteoarthritis (OA) with approximately 50% of patients reporting moderate to severe pain. Total knee replacement (TKR) is the ultimate treatment option to alleviate pain in knee OA. Nevertheless, TKR does not provide complete relief for all as approximately 20% of patients experience chronic post-operative pain. Painful peripheral stimuli may alter the central nociceptive pathways leading to central sensitisation that can influence treatment response in OA patients. Currently, there is no objective protocol for detecting whether a patient will respond to a given treatment. Therefore, there is a need for a better mechanistic understanding of individual factors affecting pain relief, consequently informing personalised treatment guidelines. The purpose of this research is to examine the feasibility of conducting a full-scale mechanistic clinical trial in painful knee OA investigating the analgesic response to intra-articular bupivacaine between those with or without evidence of central sensitisation.

Methods and analysis

The study is a feasibility, double-blinded, placebo-controlled randomised parallel study in participants with radiographically defined knee OA and with self-reported chronic knee pain. The study involves the following assessments: 1) a suite of psychometric questionnaires; 2) Quantitative Sensory Testing; 3) MRI scan of the knee and brain; 4) a 6-Minute Walk Test; and 5) an intra-articular injection of bupivacaine or placebo (sodium chloride 0.9%) into the index knee. Assessments will be repeated post intra-articular injection apart from the knee MRI scan. Our aim is to provide proof of concept and descriptive statistics to power a future mechanistic trial.

Ethics and dissemination

Ethical approval was obtained from the Health Research Authority (REC: 20/EM/0287). Results will be disseminated via peer-reviewed journals and scientific conferences. Results will also be shared with lay audiences through relevant channels, such as Pain Centre Versus Arthritis website and patient advocacy groups.

Registration details

ClinicalTrials.gov identifier: NCT05561010.

STRENGTHS AND LIMITATIONS OF THIS STUDY

- The study attempts to address a knowledge gap by providing mechanistic understanding of the factors contributing to the individual pain relief in knee OA.
- The feasibility design will support a future main trial.
- Patients were involved in the development of the study protocol. They helped to identify the most relevant outcome measures which were reordered to prioritise the patients' preferences.
- Owing to the nature of the feasibility study, no formal hypothesis testing will be performed in this trial. It will provide descriptive statistics which will inform the design and sample size requirements of a future definitive mechanistic trial.

For peer review only

INTRODUCTION

Rationale

Knee osteoarthritis (OA) is a leading cause of pain, functional disability and is a substantial economic burden on society and health care systems.[1-3] Treatment guidelines for chronic knee pain in OA are mainly focused on pain management with a combination of pharmacological and non-pharmacological treatment approaches, but no disease modifying treatment currently exists.[4] Total knee replacement (TKR) is the ultimate treatment to alleviate pain, nevertheless chronic pain is still reported in about 20% of patients after the surgery.[5] Currently there is no objective protocol to detect how a patient will respond to a given OA treatment. Moreover, there is also a poor correlation between the severity of joint damage and severity of pain[6] which points to the contribution of central pain mechanisms.

Painful peripheral stimuli may alter the central nociceptive pathways leading to central sensitisation that can influence treatment response in OA patients.[7] Sensitisation, which plays a key role in augmenting OA pain, is defined as a modified perception of pain due to increased impulses from peripheral nervous system (peripheral sensitization) along with augmentation of pain signals in the central nervous system (central sensitization).[8] Central sensitization was estimated to be present in about 30% of patients with OA[9] and was found to be a predictor for developing chronic post-operative pain after TKR.[10 11]

Validated proxies, including questionnaires, neuroimaging and quantitative sensory testing (QST), have been used to examine pain sensitization. However, a gold standard to diagnose pain sensitization in a clinical setting is lacking, and individualised mechanism-based treatment in knee OA remains a pressing need.

Evidence gap

Given the necessity for better understanding of the mechanisms of pain relief in knee OA, the purpose of this research is to examine the feasibility of conducting a full-scale mechanistic clinical trial investigating the analgesic response in knee OA between those with or without evidence of central sensitisation. The use of intra-articular bupivacaine, as an experimental medicine approach, has been shown to significantly reduce knee pain one hour after injection in previous studies[12 13]; as it temporarily blocks peripheral pain stimuli thereby potentially unmasking central pain components. Additionally, the use of comprehensive pain phenotyping tools including knee and brain MRI will enable a better mechanistic understanding of individual factors affecting pain relief, consequently informing future personalised treatment guidelines.

For peer review only

Objectives

- The primary objective of UP-KNEE study is to evaluate the feasibility of a main definitive trial by:
    - collecting data to inform sample size calculation;
    - testing the recruitment process;
    - timing of the outcome measures;
    - testing the robustness of randomisation;
    - testing the integrity of the research protocol; and
    - qualitative assessment of the acceptability of the methods of the feasibility study.
- The secondary objective of the study is to explore correlations of the analgesic response with indicators of central and peripheral pain mechanisms derived from brain and knee MRI, questionnaires and QST.

Hypothesis

For the future main trial, the hypothesis is that the analgesic response to a peripherally-targeted intervention aiming to reduce knee pain will reveal two groups. The non-responders group will be patients with predominantly centrally driven pain characteristics, who will show less analgesic response to the peripherally-targeted intervention while the responders group will be patients with predominantly peripheral pain who will show a greater analgesic response to the peripherally-targeted intervention. Both groups will respond similarly to placebo. However, this feasibility study is not intended to test the hypothesis of the main trial.

METHODS AND ANALYSIS

Study design

The study will be a feasibility, double-blinded, placebo-controlled randomised parallel study in participants with radiographically defined knee OA and with self-reported chronic knee pain. The study design was guided by the recommendations of the Consolidated Standards of Reporting Trials (CONSORT) 2010 statement extension to randomised pilot and feasibility trials.[14]

Study setting

This is a single centre study. The study activities will be carried out at the Sir Peter Mansfield Imaging Centre, University of Nottingham.

For peer review only

Sample size

This is a feasibility study aiming to provide first proof of concept and descriptive statistics to power a future diagnostic trial. We aim to recruit 50 participants (25 to each group). This would be a large enough sample to assess the feasibility and inform a main trial based on statistical methodological papers providing recommendations about sample size requirements for feasibility and pilot studies.[15-17]

Eligibility criteria

The inclusion criteria are; patients aged 45 years and older who have the capacity to give informed consent and have radiographically defined OA knee changes (Kellgren and Lawrence Grading System K/L > 2/4) with knee pain, particularly self-reported knee pain at the most severely affected side measuring between 30 mm to 80 mm on a 100 mm visual analogue scale (VAS) for rest, use or night pain, and being able to perform the six-minute walk test. The VAS anchors are: 0 as 'no pain' and 100 as 'the worst pain imaginable'.

The exclusion criteria are; patients having major medical, neurological and psychiatric co-morbidities that would preclude completion of the study protocol. Patients who have a diagnosis of osteoarthritis in any joint other than the knee with pain VAS ≥ 30 mm, fibromyalgia, systemic or local knee infection, severe coagulopathy or taking anti-coagulant therapy, known hypersensitivity to bupivacaine, taking neuropathic pain medications for their OA-related pain such as strong opioid analgesics and antiepileptic drugs will also be excluded. Known contraindications for MRI are also exclusion criteria.

Recruitment

The trial flow is summarised in Figure 1. Eligible participants will be recruited mainly through the secondary care pathway at the Trauma and Orthopaedic Department in Nottingham University Hospitals (NUH) NHS Trust. The clinical team directly involved in patient care will identify participants as part of their routine clinical review. In addition, there are other potential methods of patient recruitment to UP-KNEE study, such as Primary Care pathways and participants who have previously participated in previous research in the Pain Centre Versus Arthritis and gave consent to be contacted again regarding future studies.

Informed, written consent will be obtained prior to any research activities. The participant will be given the opportunity to choose whether they consent via phone or at the appointment. Verbal consent will be taken in accordance with NUH verbal consenting guidance. Participants can withdraw from the study at any timepoint. In

For peer review only - http://bmjopen.bmj.com/site/about/guidelines.xhtml

the event of their withdrawal, data already collected will be used, but no further data will be acquired.

Randomisation procedure and concealment of allocation

Eligible participants will be randomised in a 1:1 allocation ratio. An on-line computer service (SealedEnvelope.com) will be used for stratified randomisation to either intra-articular injection of bupivacaine or sodium chloride 0.9%. This facility generates codes that are concealed from the research team to either group. To mitigate against risk of allocation imbalance with respect to whether pain is predominantly centrally or peripherally driven, stratification will be based on the scores of CAP-Knee questionnaire which will be sent to participants before the research visit. Participants will be randomised on a 1:1 allocation basis to either bupivacaine or placebo. To ensure blinding of the investigators to the participant's group assignment, an authorised research nurse will prepare the allocated treatment. The randomisation schedule will be embedded by the research nurse in a secure password-protected folder to achieve allocation concealment.

Blinding

Investigators and participants will be blind to the treatment allocation. To reduce risk of interpretation bias, any manual image analysis will be performed by an investigator blinded to clinical status.

Experimental procedures

The study involves a single visit in which data will be collected. The interventions will include an assessment to check patients' suitability to undergo intervention with a local anaesthetic (bupivacaine) or placebo (sodium chloride 0.9%) intra-articular knee injection. During the visit, all participants will be invited to undertake the following assessments: 1) complete health questionnaires; 2) undergo QST; 3) MRI scan of the knee and brain; 4) perform a 6-Minute Walk Test; and 5) participants will then receive an intra-articular injection of bupivacaine or placebo into the index knee. Assessments will be repeated post intra-articular injection apart from the MRI scan of the knee.

The timings of the post-injection assessments were chosen based on the impact of the timing of the bupivacaine administration on its analgesic effects. As the time to peak concentration is 43.4 +/- 23.1 minute based on previous research using bupivacaine for peripheral analgesia[18], there will be a 20-minute interval post-injection to ensure sufficient time to demonstrate the full effect of the local anaesthesia and for the post-procedural care. The post-injection procedures will then be completed between 20 to 60 minutes post-injection.

For peer review only

- Psychometric questionnaires:

Questionnaires will be used to rate the pain severity and to evaluate psychosocial constructs. The questionnaires set include painDETECT[19], Pain Catastrophizing Scale (PCS)[20], the Central Aspects of Pain in the Knee (CAP-Knee) questionnaire[21], Beck Depression Inventory[22], Measure of Intermittent and Constant Osteoarthritis Pain (ICOAP)[23], EQ-5DL Health Questionnaire[24], Oxford Knee Score[25], Pittsburgh Sleep Quality Index[26], Fatigue Severity Scale and the State-Trait Anxiety Inventory.

At the end of the research visit, the participant will also be asked to fill in a custom-made questionnaire with open-ended questions intended to further investigate aspects of study feasibility and acceptability of the research methods.

- 6-Minute Walk Test (6MWT) pre and post injection of bupivacaine/placebo:

The self-paced 6MWT is aimed to be the functional outcome measure as it can assess the submaximal level of functional capacity. Participants will be requested to undertake a paced walk and are also allowed to stop and rest during performing the test. Participants will be asked to rate the level of knee pain 'pain this instant' before and immediately after the test finishes using the 0-100 VAS. The walking course of the 6MWT will be 30 metres in length with a mark every 3 metres.

- QST:

QST is a psychophysical technique evaluating sensory response to standardised mechanical or thermal stimuli. The study will use static pain parameters; pressure pain detection threshold (PPT), and dynamic parameters; temporal summation of pain (TSP) and conditioned pain modulation (CPM). Several lines of evidence suggest that QST can be used to phenotype OA pain based on mechanisms. Enrolled patients will undergo the following QST measures: PPT, TSP and CPM, which will be performed according to a standardised methodology[27-29] (Supplementary A).

- Intra-articular knee joint injection with bupivacaine/placebo:

Bupivacaine or sodium chloride 0.9% will be administered via the intra-articular route by a member of the research team (a qualified medic) with appropriate competence in intra-articular injection and the recognition and management of the potential adverse effects. The injection will be carried out under aseptic conditions.

For intra-articular injection, the injected volume of either bupivacaine 0.25% (5 ml) or sodium chloride 0.9% (5 ml) will be the same for each participant. Bupivacaine was selected for the intra-articular injection to achieve analgesia because this was shown in previous studies to significantly reduce knee pain in participants with knee OA one hour after injection.[12 13]

For peer review only

- Knee MRI before the injection:

The participants will also undergo a short structural knee MRI at 3 Tesla in order to define synovitis, bone marrow lesions, and other structural pathologies in the knee joint. Knee MRI will be evaluated using the validated semiquantitative Magnetic Resonance Imaging Osteoarthritis Knee Score (MOAKS).[30]

- Brain imaging:

The participants will undergo a multimodal MRI scan of the brain at 3 Tesla before and after the intra-articular injection. This study will use the functional magnetic resonance imaging (fMRI) protocol from studies in pain imaging that can characterise spontaneous chronic pain.[31-33]

Outcomes

Primary outcome measures

1. The change in pain score using a 100 mm Visual Analogue Scale during the six-minute walk test from baseline to one hour after intra-articular injection with bupivacaine or placebo.

2. The change in pain score using the Visual Analogue Scale at rest from baseline to one hour after intra-articular injection with bupivacaine or placebo.

Secondary outcome measures

1. QST: the change scores of PPT, TSP and CPM from baseline to post intra-articular injection with bupivacaine or placebo.

2. fMRI of the brain: the change in brain network activity from baseline to post intra-articular injection with bupivacaine or placebo.

3. Magnetic Resonance Imaging Knee Osteoarthritis Score (MOAKS)[30]: the level of joint damage quantified by MOAKS and measured at baseline.

4. Feasibility assessment: The number and percentage of eligible participants who are recruited and randomised to the study from the date of recruitment opening until the date of recruitment closing, as well as protocol completion rates and missing data rates.

5. Feasibility assessment: Evaluation of effective randomisation of participants to the study arms using a study-specific checklist and assessment of the randomisation protocol throughout the study.

For peer review only

6. Feasibility assessment: A study-specific questionnaire will be administered to participants to assess the acceptability of the study at the end of the research visit.

Data management

The collection, storage, processing and disclosure of personal information, data collection and management will comply with the requirements of the General Data Protection Regulation (GDPR) 2018. Handling of data will follow the policies and the procedures of the Sponsor (Nottingham University Hospitals NHS Trust) and the University of Nottingham. All personal data will be anonymised, and all further data analysis will be done without any reference to personal identifiable participant data.

Statistical methods

1. Considering the feasibility nature of the study, no formal hypothesis tests will be performed to statistically compare the two study arms, and descriptive statistics will be presented. Appropriate parametric or non-parametric statistics will be used according to the data characteristics.
2. Summary statistics will be used to evaluate feasibility objectives such as the feasibility to recruit, timing measurements, to describe the sample and to inform the future main trial by providing power and sample size calculation. Participant acceptability feedback will be qualitatively synthesised using thematic analysis.
3. Primary and secondary outcomes will be descriptively summarised by group as follows:
    a. Correlations between the change of pain score and QST measurements at baseline and after intra-articular injection with bupivacaine or placebo.
    b. Correlations between the change of pain score and brain network activity at baseline and after intra-articular injection with bupivacaine or placebo using predefined seeds in the pain processing regions.
    c. Correlations between the change of pain score and the level of joint damage quantified by MOAKS and measured at baseline.
4. Estimates of between-group effect will be reported as estimates with 95% confidence intervals without *p* values.
5. In view of the exploratory nature of the study, missing data will not be imputed. The proportion of missing data for each outcome will be described.
6. Differences in effects will be reported descriptively for primary and secondary outcomes between the following subgroups:
    a. Based on the stratification variable according to CAP-Knee questionnaire scores with respect to whether pain is predominantly centrally or peripherally driven.
    b. The patients will be divided into responders and non-responders based on a cut-off value of ≥ 17 mm reduction in VAS post- injection.[34]

For peer review only

Safety and adverse events

The study was deemed a low-risk study by the Sponsor (Nottingham University Hospitals NHS Trust). Adverse events will be recorded and reported according to the policies of the Local Research Ethics Committee and Nottingham University Hospitals NHS Trust.[35]

Patient and public involvement

The study protocol and documents have been re-shaped according to input received in two patient and public involvement (PPI) events via Pain Centre Versus Arthritis PPI advisory group. This advisory group was composed of people with OA who provided constructive feedback on the outcome measures and the study methods which led to enhanced study design.

ETHICS AND DISSEMINATION

The study has been approved by the Nottingham Research Ethics Committee 1 (REC Reference: 20/EM/0287). The study results will be disseminated through peer-reviewed journals and communicated at scientific meetings and conferences. Results will also be presented in relevant patient websites. Authorship eligibility will be based on the recommendations from the International Committee of Medical Journal Editors (ICMJE).

PERSPECTIVES OF THE STUDY

This feasibility RCT will provide first proof of concept to a future main trial. The study has the scope to enhance the understanding of knee OA pain mechanisms and to pave the way for individualised treatment in knee OA.

CURRENT TRIAL STATUS

HRA approval has been obtained. Recruitment was initiated in late 2022.

FOOTNOTES

- **Authors' contributions:** state how each author was involved in writing the protocol.

BS conceived the study and designed the protocol with YZ, RK, DC, TK, DAW and DA. YZ communicated with the Regulatory Authorities, initiated the study and wrote the first draft of the manuscript. All authors reviewed and approved this manuscript for submission.

- **Funding statement:**

This project is supported by Versus Arthritis (Grant reference number: 20777).

- **Competing interests statement:**

For peer review only

None declared.

REFERENCES

1. Hunter DJ, Bierma-Zeinstra S. Osteoarthritis. *The Lancet* 2019;393(10182):1745-59. doi: https://doi.org/10.1016/S0140-6736(19)30417-9

2. Quicke JG, Conaghan PG, Corp N, et al. Osteoarthritis year in review 2021: epidemiology & therapy. *Osteoarthr Cartilage* 2022;30(2):196-206. doi: https://doi.org/10.1016/j.joca.2021.10.003

3. Jackson J, Iyer R, Mellor J, et al. The Burden of Pain Associated with Osteoarthritis in the Hip or Knee from the Patient's Perspective: A Multinational Cross-Sectional Study. *Advances in therapy* 2020;37(9):3985-99. doi: 10.1007/s12325-020-01445-4 [published Online First: 2020/07/31]

4. Makarczyk MJ, Gao Q, He Y, et al. Current Models for Development of Disease-Modifying Osteoarthritis Drugs. *Tissue engineering Part C, Methods* 2021;27(2):124-38. doi: 10.1089/ten.TEC.2020.0309 [published Online First: 2021/01/07]

5. Wylde V, Beswick A, Bruce J, et al. Chronic pain after total knee arthroplasty. *EFORT open reviews* 2018;3(8):461-70. doi: 10.1302/2058-5241.3.180004

6. Finan PH, Buenaver LF, Bounds SC, et al. Discordance between pain and radiographic severity in knee osteoarthritis: findings from quantitative sensory testing of central sensitization. *Arthritis and rheumatism* 2013;65(2):363-72. doi: 10.1002/art.34646

7. Arendt-Nielsen L. Pain sensitisation in osteoarthritis. *Clinical and experimental rheumatology* 2017;35 Suppl 107(5):68-74. [published Online First: 2017/10/03]

8. Arendt-Nielsen L, Skou ST, Nielsen TA, et al. Altered Central Sensitization and Pain Modulation in the CNS in Chronic Joint Pain. *Current osteoporosis reports* 2015;13(4):225-34. doi: 10.1007/s11914-015-0276-x [published Online First: 2015/06/01]

9. Lluch E, Torres R, Nijs J, et al. Evidence for central sensitization in patients with osteoarthritis pain: A systematic literature review. *European Journal of Pain* 2014;18(10):1367-75. doi: 10.1002/j.1532-2149.2014.499.x

10. Skou ST, Graven-Nielsen T Fau - Rasmussen S, Rasmussen S Fau - Simonsen OH, et al. Facilitation of pain sensitization in knee osteoarthritis and persistent post-operative pain: a cross-sectional study. (1532-2149 (Electronic))

11. Kurien T, Arendt-Nielsen L, Petersen KK, et al. Preoperative Neuropathic Pain-like Symptoms and Central Pain Mechanisms in Knee Osteoarthritis Predicts Poor

For peer review only

Outcome 6 Months After Total Knee Replacement Surgery. *The Journal of Pain* 2018;19(11):1329-41. doi: https://doi.org/10.1016/j.jpain.2018.05.011

12. Creamer P, Hunt M, Dieppe P. Pain mechanisms in osteoarthritis of the knee: effect of intraarticular anesthetic. *The Journal of rheumatology* 1996;23(6):1031-6. [published Online First: 1996/06/01]
13. Hassan BS. Effect of pain reduction on postural sway, proprioception, and quadriceps strength in subjects with knee osteoarthritis. *Annals of the Rheumatic Diseases* 2002;61(5):422-28. doi: 10.1136/ard.61.5.422
14. Eldridge SM, Chan CL, Campbell MJ, et al. CONSORT 2010 statement: extension to randomised pilot and feasibility trials. *BMJ* 2016;355:i5239. doi: 10.1136/bmj.i5239
15. Sim J, Lewis M. The size of a pilot study for a clinical trial should be calculated in relation to considerations of precision and efficiency. *Journal of clinical epidemiology* 2012;65(3):301-8. doi: 10.1016/j.jclinepi.2011.07.011 [published Online First: 2011/12/16]
16. Billingham SA, Whitehead Al Fau - Julious SA, Julious SA. An audit of sample sizes for pilot and feasibility trials being undertaken in the United Kingdom registered in the United Kingdom Clinical Research Network database. (1471-2288 (Electronic))
17. Whitehead AL, Julious SA, Cooper CL, et al. Estimating the sample size for a pilot randomised trial to minimise the overall trial sample size for the external pilot and main trial for a continuous outcome variable. (1477-0334 (Electronic))
18. Katz JA, Kaeding CS, Hill JR, et al. The pharmacokinetics of bupivacaine when injected intra-articularly after knee arthroscopy. *Anesthesia and analgesia* 1988;67(9):872-5. [published Online First: 1988/09/01]
19. Freynhagen R, Baron R, Gockel U, et al. painDETECT: a new screening questionnaire to identify neuropathic components in patients with back pain. *Current medical research and opinion* 2006;22(10):1911-20. doi: 10.1185/030079906x132488 [published Online First: 2006/10/07]
20. Sullivan MJL, Bishop SR, Pivik J. The Pain Catastrophizing Scale: Development and validation. *Psychological Assessment* 1995;7(4):524-32. doi: 10.1037/1040-3590.7.4.524
21. Akin-Akinyosoye K, James RJE, McWilliams DF, et al. The Central Aspects of Pain in the Knee (CAP-Knee) questionnaire; a mixed-methods study of a self-report instrument for assessing central mechanisms in people with knee pain. (1522-9653 (Electronic))
22. Beck At Fau - Ward CH, Ward Ch Fau - Mendelson M, Mendelson M Fau - Mock J, et al. An inventory for measuring depression. (0003-990X (Print))

For peer review only

23. Hawker GA, Davis AM, French MR, et al. Development and preliminary psychometric testing of a new OA pain measure – an OARSI/OMERACT initiative. *Osteoarthritis and cartilage* 2008;16(4):409-14. doi: https://doi.org/10.1016/j.joca.2007.12.015
24. Herdman M, Gudex C, Lloyd A, et al. Development and preliminary testing of the new five-level version of EQ-5D (EQ-5D-5L). *Quality of Life Research* 2011;20(10):1727-36. doi: 10.1007/s11136-011-9903-x
25. Dawson J, Fitzpatrick R, Murray D, et al. Questionnaire on the perceptions of patients about total knee replacement. *The Journal of Bone and Joint Surgery British volume* 1998;80-B(1):63-69. doi: 10.1302/0301-620X.80B1.0800063
26. Smyth C. The Pittsburgh Sleep Quality Index (PSQI). (0098-9134 (Print))
27. Rathleff MS, Petersen KK, Arendt-Nielsen L, et al. Impaired Conditioned Pain Modulation in Young Female Adults with Long-Standing Patellofemoral Pain: A Single Blinded Cross-Sectional Study. *Pain medicine (Malden, Mass)* 2016;17(5):980-8. doi: 10.1093/pm/pnv017 [published Online First: 2016/01/28]
28. Vaegter HB, Graven-Nielsen T. Pain modulatory phenotypes differentiate subgroups with different clinical and experimental pain sensitivity. *Pain* 2016;157(7):1480-8. doi: 10.1097/j.pain.0000000000000543 [published Online First: 2016/03/11]
29. Petersen KK, Arendt-Nielsen L, Finocchietti S, et al. Age Interactions on Pain Sensitization in Patients With Severe Knee Osteoarthritis and Controls. *The Clinical journal of pain* 2017;33(12):1081-87. doi: 10.1097/AJP.0000000000000495 [published Online First: 2017/03/10]
30. Hunter DJ, Guermazi A, Lo GH, et al. Evolution of semi-quantitative whole joint assessment of knee OA: MOAKS (MRI Osteoarthritis Knee Score). *Osteoarthritis and cartilage* 2011;19(8):990-1002. doi: 10.1016/j.joca.2011.05.004 [published Online First: 2011/06/08]
31. Cottam WJ, Iwabuchi SJ, Drabek MM, et al. Altered connectivity of the right anterior insula drives the pain connectome changes in chronic knee osteoarthritis. *Pain* 2018;159(5):929-38. doi: 10.1097/j.pain.0000000000001209 [published Online First: 2018/03/21]
32. Iwabuchi SJ, Xing Y, Cottam WJ, et al. Brain perfusion patterns are altered in chronic knee pain: a spatial covariance analysis of arterial spin labelling MRI. *Pain* 2020 doi: 10.1097/j.pain.0000000000001829 [published Online First: 2020/02/19]
33. Drabek MM, Cottam WJ, Iwabuchi SJ, et al. Prescription opioid-related alterations to amygdalar and thalamic functional networks in chronic knee pain: A retrospective case control resting-state connectivity study. *medRxiv* 2022:2022.03.01.22271607. doi: 10.1101/2022.03.01.22271607

For peer review only

34. Bird SB, Dickson EW. Clinically significant changes in pain along the visual analog scale. *Annals of Emergency Medicine* 2001;38(6):639-43. doi: 10.1067/mem.2001.118012
35. Adverse Event Reporting SOP 2022 [Internet]. Nottingham University Hospitals NHS Trust. Available from: https://www.nuh.nhs.uk/guidance-researchers?search-media=adverse%2Bevents [accessed 2022Oct3].

For peer review only

Figure legends:

Figure 1: Overall trial flow

Key: TKR - total knee replacement, OA - osteoarthritis, PIS - participant information sheet, SPMIC - Sir Peter Mansfield Imaging Centre, QMC - Queen's Medical Centre, QST - quantitative sensory testing, fMRI - functional magnetic resonance imaging.

For peer review only

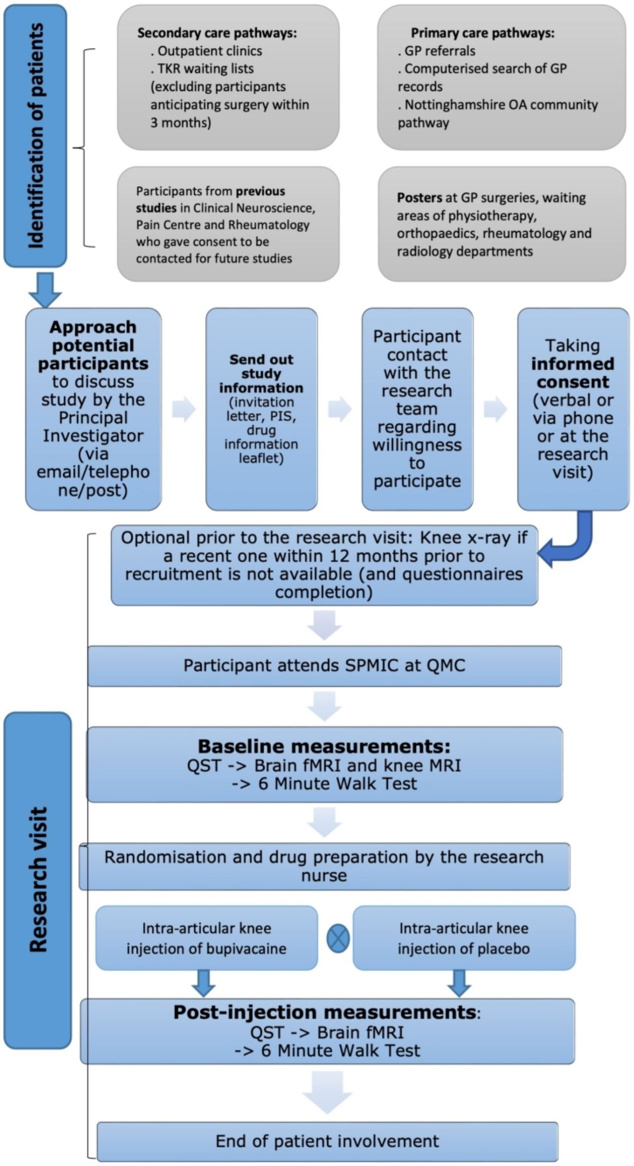

Figure 1: Overall trial flow
Key: TKR - total knee replacement, OA - osteoarthritis, PIS - participant information sheet, SPMIC - Sir Peter Mansfield Imaging Centre, QMC - Queen's Medical Centre, QST - quantitative sensory testing, fMRI - functional magnetic resonance imaging.

175x230mm (300 x 300 DPI)

Supplementary A:

Quantitative Sensory Testing (QST) protocol:
This protocol follows standardised methodology described in previous studies.[1–5]

Pressure Algometry
To examine pressure pain thresholds (PPT), a handheld pressure algometer (Somedic AB, Sösdala, Sweden) will be used. Pressure will be increased by 30kPa/s until the participant perceives a change of the stimulus from a pressure sensation to pain sensation. The participant will then press a response button and the algometer probe will be immediately withdrawn. The PPT score in kPa is the pressure applied at the time the response button was pressed.
PPT will be recorded from the following sites:
1. Medial joint line of the index knee (the most painful knee): 2 cm proximal to superomedial patellar margin.
2. Lateral joint line of the index knee: 2 cm proximal to superolateral patellar margin.
3. The tibialis anterior muscle as a distant site to examine spreading sensitization.
2. the extensor carpi radialis longus (ECRL) muscle as a remote site to examine widespread hyperalgesia.
A practise test will be done on each site then PPTs will be recorded in triplicates which will then be averaged per site for further analysis.

Cuff Pressure Algometry
A cuff algometer (NociTech and Aalborg University, Denmark) comprised of two 13 cm single chamber tourniquet cuffs (VBM, Sulz, Germany) will use computer-controlled cuff pressure stimuli to assess pain sensitivity of the deep tissue. The device is connected to an air compressor and an electronic VAS rating system (Aalborg University) which is sampled at 10 Hz.
the cuff will be applied at the level of the ipsilateral gastrocnemius muscle and will be inflated at a rate of 1kPa/s until max pressure limit of 100 kPa. The participant will rate his pressure-induced pain severity continuously using the VAS rating system and will press a button to release the pressure. The cuff pain detection threshold (PDT) is the pressure value when the subject rated pain as 1 cm on the VAS rating system.
The cuff pain tolerance threshold (PTT) is the value of maximum pressure at the point the subject had to press the release button because of intolerable pain severity.

Temporal Summation of Pain (TSP) by Cuff Algometry
Ten consecutive cuff pressure stimulations comprised of 1-second stimulus with a 2-second inter-stimulus-interval with an intensity equal to the PTT score will be applied ipsilateral to the index knee. Participants will rate the pain severity continuously throughout the stimulation using the VAS slider and to not return the VAS slider to zero in between cuff stimulations. A constant pressure of 5 kPa is retained by the cuff in-between stimulations to ensure that fixed position of the cuff on the leg during the examination. The VAS score is recorded immediately after each individual cuff stimulus.
TSP score is calculated by subtracting the mean VAS score of the first to 4th cuff stimulations (VAS-I) from the mean VAS score of the 8th to 10th cuff stimulations (VAS-II).

For peer review only

Conditioning Pain Modulation by Cuff Algometry

A painful conditioning stimulus will be applied contralateral to the index knee using the cuff with an inflation pressure equals 70% of the participant's cuff PTT. As a test stimulus, PDT will be simultaneously examined on the ipsilateral side using the second cuff. Conditioning pain modulation (CPM) will be calculated by subtracting the unconditioned PDT score from the conditioned PDT score.

References

1. Petersen KK, Arendt-Nielsen L, Finocchietti S, et al. Age Interactions on Pain Sensitization in Patients With Severe Knee Osteoarthritis and Controls. *Clin J Pain*. 2017;33(12):1081-1087. doi:10.1097/AJP.0000000000000495
2. Rathleff MS, Petersen KK, Arendt-Nielsen L, Thorborg K, Graven-Nielsen T. Impaired Conditioned Pain Modulation in Young Female Adults with Long-Standing Patellofemoral Pain: A Single Blinded Cross-Sectional Study. *Pain Med*. 2016;17(5):980-988. doi:10.1093/pm/pnv017
3. Vaegter HB, Graven-Nielsen T. Pain modulatory phenotypes differentiate subgroups with different clinical and experimental pain sensitivity. *Pain*. 2016;157(7):1480-1488. doi:10.1097/j.pain.0000000000000543
4. Kurien T, Arendt-Nielsen L, Petersen KK, Graven-Nielsen T, Scammell BE. Preoperative Neuropathic Pain-like Symptoms and Central Pain Mechanisms in Knee Osteoarthritis Predicts Poor Outcome 6 Months After Total Knee Replacement Surgery. *J Pain*. 2018;19(11):1329-1341. doi:https://doi.org/10.1016/j.jpain.2018.05.011
5. Iwabuchi SJ, Xing Y, Cottam WJ, et al. Brain perfusion patterns are altered in chronic knee pain: a spatial covariance analysis of arterial spin labelling MRI. *Pain*. 2020;161(6):1255-1263. doi:10.1097/j.pain.0000000000001829

For peer review only

BMJ Open

Is there a difference in the analgesic response to intra-articular bupivacaine injection in people with knee osteoarthritis pain with or without central sensitisation?: protocol of a feasibility randomised controlled trial

Journal:	*BMJ Open*
Manuscript ID	bmjopen-2023-072138.R1
Article Type:	Protocol
Date Submitted by the Author:	25-May-2023
Complete List of Authors:	Zedan, Yasmine; University of Nottingham, Radiological Sciences, Mental Health & Clinical Neurosciences, School of Medicine; University of Nottingham, Pain Centre Versus Arthritis, School of Medicine<br>Knaggs, Roger; University of Nottingham, Clinical Pharmacy Practice, School of Pharmacy<br>Cooper, Dale; Keele University, School of Allied Health Professions<br>Kurien, Thomas ; University of Nottingham, Pain Centre Versus Arthritis, School of Medicine; University of Nottingham, Academic Orthopaedics, Trauma and Sports Medicine, School of Medicine<br>Walsh, David; University of Nottingham, Pain Centre Versus Arthritis, School of Medicine; University of Nottingham, Academic Rheumatology, School of Medicine<br>Auer, Dorothee; University of Nottingham, Radiological Sciences, Mental Health & Clinical Neurosciences, School of Medicine; University of Nottingham, Pain Centre Versus Arthritis, School of Medicine<br>Scammell, Brigitte; University of Nottingham, Pain Centre Versus Arthritis, School of Medicine; University of Nottingham, Academic Orthopaedics, Trauma and Sports Medicine, School of Medicine
**Primary Subject Heading**:	Patient-centred medicine
Secondary Subject Heading:	Rheumatology, Radiology and imaging
Keywords:	Knee < ORTHOPAEDIC & TRAUMA SURGERY, Musculoskeletal disorders < ORTHOPAEDIC & TRAUMA SURGERY, Protocols & guidelines < HEALTH SERVICES ADMINISTRATION & MANAGEMENT, Magnetic resonance imaging < RADIOLOGY & IMAGING

SCHOLARONE™
Manuscripts

Is there a difference in the analgesic response to intra-articular bupivacaine injection in people with knee osteoarthritis pain with or without central sensitisation?: protocol of a feasibility randomised controlled trial

For peer review only

ABSTRACT

Introduction

Pain is the main symptom of osteoarthritis (OA) with approximately 50% of patients reporting moderate to severe pain. Total knee replacement (TKR) is the ultimate treatment option to alleviate pain in knee OA. Nevertheless, TKR does not provide complete relief for all as approximately 20% of patients experience chronic post-operative pain. Painful peripheral stimuli may alter the central nociceptive pathways leading to central sensitisation that can influence treatment response in OA patients. Currently, there is no objective protocol for detecting whether a patient will respond to a given treatment. Therefore, there is a need for a better mechanistic understanding of individual factors affecting pain relief, consequently informing personalised treatment guidelines. The purpose of this research is to examine the feasibility of conducting a full-scale mechanistic clinical trial in painful knee OA investigating the analgesic response to intra-articular bupivacaine between those with or without evidence of central sensitisation.

Methods and analysis

The study is a feasibility, double-blinded, placebo-controlled randomised parallel study in participants with radiographically defined knee OA and with self-reported chronic knee pain. The study involves the following assessments: 1) a suite of psychometric questionnaires; 2) Quantitative Sensory Testing; 3) MRI scan of the knee and brain; 4) a 6-Minute Walk Test; and 5) an intra-articular injection of bupivacaine or placebo (sodium chloride 0.9%) into the index knee. Assessments will be repeated post intra-articular injection apart from the knee MRI scan. Our aim is to provide proof of concept and descriptive statistics to power a future mechanistic trial.

Ethics and dissemination

Ethical approval was obtained from the Health Research Authority (REC: 20/EM/0287). Results will be disseminated via peer-reviewed journals and scientific conferences. Results will also be shared with lay audiences through relevant channels, such as Pain Centre Versus Arthritis website and patient advocacy groups.

Registration details

ClinicalTrials.gov identifier: NCT05561010.

STRENGTHS AND LIMITATIONS OF THIS STUDY

- The study attempts to address a knowledge gap by providing mechanistic understanding of the factors contributing to the individual pain relief in knee OA.
- The feasibility design will support a future main trial.
- Patients were involved in the development of the study protocol. They helped to identify the most relevant outcome measures which were reordered to prioritise the patients' preferences.
- Owing to the nature of the feasibility study, no formal hypothesis testing will be performed in this trial. It will provide descriptive statistics which will inform the design and sample size requirements of a future definitive mechanistic trial.

For peer review only

INTRODUCTION

Rationale

Knee osteoarthritis (OA) is a leading cause of pain, functional disability and is a substantial economic burden on society and health care systems.[1-3] Treatment guidelines for chronic knee pain in OA are mainly focused on pain management with a combination of pharmacological and non-pharmacological treatment approaches, but no disease modifying treatment currently exists.[4] Total knee replacement (TKR) is the ultimate treatment to alleviate pain, nevertheless chronic pain is still reported in about 20% of patients after the surgery.[5] Currently there is no objective protocol to detect how a patient will respond to a given OA treatment. Moreover, there is also a poor correlation between the severity of joint damage and severity of pain[6] which points to the contribution of central pain mechanisms.

Painful peripheral stimuli may alter the central nociceptive pathways leading to central sensitisation that can influence treatment response in OA patients.[7] Sensitisation, which plays a key role in augmenting OA pain, is defined as a modified perception of pain due to increased impulses from peripheral nervous system (peripheral sensitization) along with augmentation of pain signals in the central nervous system (central sensitization).[8] Central sensitization was estimated to be present in about 30% of patients with OA[9] and was found to be a predictor for developing chronic post-operative pain after TKR.[10 11]

Validated proxies, including questionnaires, neuroimaging and quantitative sensory testing (QST), have been used to examine pain sensitization. However, a gold standard to diagnose pain sensitization in a clinical setting is lacking, and individualised mechanism-based treatment in knee OA remains a pressing need.

Evidence gap

Given the necessity for better understanding of the mechanisms of pain relief in knee OA, the purpose of this research is to examine the feasibility of conducting a full-scale mechanistic clinical trial investigating the analgesic response in knee OA between those with or without evidence of central sensitisation. The use of intra-articular bupivacaine, as an experimental medicine approach, has been shown to significantly reduce knee pain one hour after injection in previous studies[12 13]; as it temporarily blocks peripheral pain stimuli thereby potentially unmasking central pain components. Given that peripheral input commonly triggers central sensitization, the theory that local anaesthesia will assist in differentiating patients with centrally mediated pain mechanisms is also supported by the notion that regional anaesthesia or local anaesthesia can reduce the risk of persistent postoperative pain six months after surgery according to a previous systematic review and meta-analysis.[14] Additionally, the use of comprehensive pain phenotyping tools including knee and

For peer review only

brain MRI will enable a better mechanistic understanding of individual factors affecting pain relief, consequently informing future personalised treatment guidelines.

Objectives

- The primary objective of UP-KNEE study is to evaluate the feasibility of a main definitive trial by:
  - collecting data to inform sample size calculation;
  - testing the recruitment process;
  - timing of the outcome measures;
  - testing the robustness of randomisation;
  - testing the integrity of the research protocol; and
  - qualitative assessment of the acceptability of the methods of the feasibility study.
- The secondary objective of the study is to explore correlations of the analgesic response with indicators of central and peripheral pain mechanisms derived from brain and knee MRI, questionnaires and QST.

Hypothesis

For the future main trial, the hypothesis is that the analgesic response to a peripherally-targeted intervention aiming to reduce knee pain will reveal two groups. The non-responders group will be patients with predominantly centrally driven pain characteristics, who will show less analgesic response to the peripherally-targeted intervention while the responders group will be patients with predominantly peripheral pain who will show a greater analgesic response to the peripherally-targeted intervention. Both groups will respond similarly to placebo. However, this feasibility study is not intended to test the hypothesis of the main trial.

METHODS AND ANALYSIS

Study design

The study will be a feasibility, double-blinded, placebo-controlled randomised parallel study in participants with radiographically defined knee OA and with self-reported chronic knee pain. The study design was guided by the recommendations of the Consolidated Standards of Reporting Trials (CONSORT) 2010 statement extension to randomised pilot and feasibility trials.[15] The study protocol has been reported in accordance with the Standard Protocol Items: Recommendations for Interventional Trials (SPIRIT) guidelines (Supplementary A).[16]

For peer review only

Study setting

This is a single centre study. The study activities will be carried out at the Sir Peter Mansfield Imaging Centre, University of Nottingham.

Sample size

This is a feasibility study aiming to provide first proof of concept and descriptive statistics to power a future diagnostic trial. We aim to recruit 50 participants (25 to each group). This would be a large enough sample to assess the feasibility and inform a main trial based on statistical methodological papers providing recommendations about sample size requirements for feasibility and pilot studies.[17-19]

Eligibility criteria

The inclusion criteria are; patients aged 45 years and older who have the capacity to give informed consent and have radiographically defined OA knee changes (Kellgren and Lawrence Grading System K/L > 2/4) with knee pain, particularly self-reported knee pain at the most severely affected side measuring between 30 mm to 80 mm on a 100 mm visual analogue scale (VAS) for rest, use or night pain, and being able to perform the six-minute walk test. The VAS anchors are: 0 as 'no pain' and 100 as 'the worst pain imaginable'.

The exclusion criteria are; patients having major medical, neurological and psychiatric co-morbidities that would preclude completion of the study protocol. Patients who have a diagnosis of osteoarthritis in any joint other than the knee with pain VAS $\geq$ 30 mm, fibromyalgia, systemic or local knee infection, severe coagulopathy or taking anti-coagulant therapy, known hypersensitivity to bupivacaine, taking neuropathic pain medications for their OA-related pain such as strong opioid analgesics and antiepileptic drugs will also be excluded. Known contraindications for MRI are also exclusion criteria.

Recruitment

The trial flow is summarised in Figure 1. Eligible participants will be recruited mainly through the secondary care pathway at the Trauma and Orthopaedic Department in Nottingham University Hospitals (NUH) NHS Trust. The clinical team directly involved in patient care will identify participants as part of their routine clinical review. In addition, there are other potential methods of patient recruitment to UP-KNEE study, such as Primary Care pathways and participants who have previously participated in previous research in the Pain Centre Versus Arthritis and gave consent to be contacted again regarding future studies.

For peer review only

Informed, written consent will be obtained prior to any research activities (Supplementary B). The participant will be given the opportunity to choose whether they consent via phone or at the appointment. Verbal consent will be taken in accordance with NUH verbal consenting guidance. Participants can withdraw from the study at any timepoint. In the event of their withdrawal, data already collected will be used, but no further data will be acquired.

Randomisation procedure and concealment of allocation

Eligible participants will be randomised in a 1:1 allocation ratio. An on-line computer service (SealedEnvelope.com) will be used for stratified randomisation to either intra-articular injection of bupivacaine or sodium chloride 0.9%. This facility generates codes that are concealed from the research team to either group. To mitigate against risk of allocation imbalance with respect to whether pain is predominantly centrally or peripherally driven, stratification will be based on the scores of CAP-Knee questionnaire which will be sent to participants before the research visit. Participants will be randomised on a 1:1 allocation basis to either bupivacaine or placebo. To ensure blinding of the investigators to the participant's group assignment, an authorised research nurse will prepare the allocated treatment. The randomisation schedule will be embedded by the research nurse in a secure password-protected folder to achieve allocation concealment.

Blinding

Investigators and participants will be blind to the treatment allocation. To reduce risk of interpretation bias, investigators analysing the data will also be blinded to the treatment allocation, and any manual image analysis will be performed by an investigator blinded to clinical status.

Emergency unblinding will only be done if emergencies occur that can be directly linked to the study medication, where it is essential to know if the patient was actually given bupivacaine.

Experimental procedures

The study involves a single visit in which data will be collected. As per the study protocol (version 1.6, 26Jan2023), the interventions will include an assessment to check patients' suitability to undergo intervention with a local anaesthetic (bupivacaine) or placebo (sodium chloride 0.9%) intra-articular knee injection. During the visit, all participants will be invited to undertake the following assessments: 1) complete health questionnaires; 2) undergo QST; 3) MRI scan of the knee and brain; 4) perform a 6-Minute Walk Test; and 5) participants will then receive an intra-articular injection of bupivacaine or placebo into the index knee. Assessments will be repeated post intra-articular injection apart from the MRI scan of the knee.

For peer review only

Supplementary C contains a detailed description of the study intervention, written in accordance with the Template for Intervention Description and Replication (TIDieR) checklist and guide.[20]

The timings of the post-injection assessments were chosen based on the impact of the timing of the bupivacaine administration on its analgesic effects. As the time to peak concentration is 43.4 +/- 23.1 minute based on previous research using bupivacaine for peripheral analgesia[21], there will be a 20-minute interval post-injection to ensure sufficient time to demonstrate the full effect of the local anaesthesia and for the post-procedural care. The post-injection procedures will then be completed between 20 to 60 minutes post-injection.

- Psychometric questionnaires:

Questionnaires will be used to rate the pain severity and to evaluate psychosocial constructs. The questionnaires set include painDETECT[22], Pain Catastrophizing Scale (PCS)[23], the Central Aspects of Pain in the Knee (CAP-Knee) questionnaire[24], Beck Depression Inventory[25], Measure of Intermittent and Constant Osteoarthritis Pain (ICOAP)[26], EQ-5DL Health Questionnaire[27], Oxford Knee Score[28], Pittsburgh Sleep Quality Index[29], Fatigue Severity Scale and the State-Trait Anxiety Inventory.

At the end of the research visit, the participant will also be asked to fill in a custom-made questionnaire with open-ended questions intended to further investigate aspects of study feasibility and acceptability of the research methods.

- 6-Minute Walk Test (6MWT) pre and post injection of bupivacaine/placebo:

The self-paced 6MWT is aimed to be the functional outcome measure as it can assess the submaximal level of functional capacity. Participants will be requested to undertake a paced walk and are also allowed to stop and rest during performing the test. Participants will be asked to rate the level of knee pain 'pain this instant' before and immediately after the test finishes using the 0-100 VAS. The walking course of the 6MWT will be 30 metres in length with a mark every 3 metres.

- QST:

QST is a psychophysical technique evaluating sensory response to standardised mechanical or thermal stimuli. The study will use static pain parameters; pressure pain detection threshold (PPT), and dynamic parameters; temporal summation of pain (TSP) and conditioned pain modulation (CPM). Several lines of evidence suggest that QST can be used to phenotype OA pain based on mechanisms. Enrolled patients will undergo the following QST measures: PPT, TSP and CPM, which will be performed according to a standardised methodology[30-32] (Supplementary D).

For peer review only

- Intra-articular knee joint injection with bupivacaine/placebo:

Bupivacaine or sodium chloride 0.9% will be administered via the intra-articular route by a member of the research team (a qualified medic) with appropriate competence in intra-articular injection and the recognition and management of the potential adverse effects. The injection will be carried out under aseptic conditions.

For intra-articular injection, the injected volume of either bupivacaine 0.25% (5 ml) or sodium chloride 0.9% (5 ml) will be the same for each participant. Bupivacaine was selected for the intra-articular injection to achieve analgesia because this was shown in previous studies to significantly reduce knee pain in participants with knee OA one hour after injection.[12 13]

- Knee MRI before the injection:

The participants will also undergo a short structural knee MRI at 3 Tesla in order to define synovitis, bone marrow lesions, and other structural pathologies in the knee joint. Knee MRI will be evaluated using the validated semiquantitative Magnetic Resonance Imaging Osteoarthritis Knee Score (MOAKS).[33]

- Brain imaging:

The participants will undergo a multimodal MRI scan of the brain at 3 Tesla before and after the intra-articular injection. This study will use the functional magnetic resonance imaging (fMRI) protocol from studies in pain imaging that can characterise spontaneous chronic pain.[34-36]

Outcomes

Primary outcome measures

1. The change in pain score using a 100 mm Visual Analogue Scale during the six-minute walk test from baseline to one hour after intra-articular injection with bupivacaine or placebo.

2. The change in pain score using the Visual Analogue Scale at rest from baseline to one hour after intra-articular injection with bupivacaine or placebo.

Secondary outcome measures

1. QST: the change scores of PPT, TSP and CPM from baseline to post intra-articular injection with bupivacaine or placebo.

2. fMRI of the brain: the change in brain network activity from baseline to post intra-articular injection with bupivacaine or placebo.

For peer review only

3. Magnetic Resonance Imaging Knee Osteoarthritis Score (MOAKS)[33]: the level of joint damage quantified by MOAKS and measured at baseline.

4. Feasibility assessment: The number and percentage of eligible participants who are recruited and randomised to the study from the date of recruitment opening until the date of recruitment closing, as well as protocol completion rates and missing data rates.

5. Feasibility assessment: Evaluation of effective randomisation of participants to the study arms using a study-specific checklist and assessment of the randomisation protocol throughout the study.

6. Feasibility assessment: A study-specific questionnaire will be administered to participants to assess the acceptability of the study at the end of the research visit.

Data management and auditing

The collection, storage, processing and disclosure of personal information, data collection and management will comply with the requirements of the General Data Protection Regulation (GDPR) 2018. Handling of data will follow the policies and the procedures of the Sponsor (Nottingham University Hospitals NHS Trust) and the University of Nottingham. All personal data will be anonymised, and all further data analysis will be done without any reference to personal identifiable participant data.

The trial conduct will be monitored by the Sponsor (Nottingham University Hospitals NHS Trust).

Statistical methods

1. Considering the feasibility nature of the study, no formal hypothesis tests will be performed to statistically compare the two study arms, and descriptive statistics will be presented. Appropriate parametric or non-parametric statistics will be used according to the data characteristics.
2. Summary statistics will be used to evaluate feasibility objectives such as the feasibility to recruit, timing measurements, to describe the sample and to inform the future main trial by providing power and sample size calculation. Participant acceptability feedback will be qualitatively synthesised using thematic analysis.
3. Primary and secondary outcomes will be descriptively summarised by group as follows:
    a. Correlations between the change of pain score and QST measurements at baseline and after intra-articular injection with bupivacaine or placebo.

For peer review only

   b. Correlations between the change of pain score and brain network activity at baseline and after intra-articular injection with bupivacaine or placebo using predefined seeds in the pain processing regions.
   c. Correlations between the change of pain score and the level of joint damage quantified by MOAKS and measured at baseline.
4. Estimates of between-group effect will be reported as estimates with 95% confidence intervals without *p* values.
5. In view of the exploratory nature of the study, missing data will not be imputed. The proportion of missing data for each outcome will be described.
6. Differences in effects will be reported descriptively for primary and secondary outcomes between the following subgroups:
   a. Based on the stratification variable according to CAP-Knee questionnaire scores with respect to whether pain is predominantly centrally or peripherally driven.
   b. The patients will be divided into responders and non-responders based on a cut-off value of ≥ 17 mm reduction in VAS post- injection.[37]

Safety and adverse events

The study was deemed a low-risk study by the Sponsor (Nottingham University Hospitals NHS Trust). Adverse events will be recorded and reported according to the policies of the Local Research Ethics Committee and Nottingham University Hospitals NHS Trust.[38]

Patient and public involvement

The study protocol and documents have been re-shaped according to input received in two patient and public involvement (PPI) events via Pain Centre Versus Arthritis PPI advisory group. This advisory group was composed of people with OA who provided constructive feedback on the outcome measures and the study methods which led to enhanced study design. The PPI will be maintained throughout the study and will help with dissemination of the study findings and outcomes to ensure a broader perspective.

ETHICS AND DISSEMINATION

The study has been approved by the Nottingham Research Ethics Committee 1 (REC Reference: 20/EM/0287). The study results will be disseminated through peer-reviewed journals and communicated at scientific meetings and conferences. Results will also be presented in relevant patient websites. Authorship eligibility will be based on the recommendations from the International Committee of Medical Journal Editors (ICMJE).

For peer review only

PERSPECTIVES OF THE STUDY

This feasibility RCT will provide first proof of concept to a future main trial. The study has the scope to enhance the understanding of knee OA pain mechanisms and to pave the way for individualised treatment in knee OA.

CURRENT TRIAL STATUS

HRA approval has been obtained. Recruitment was initiated in late 2022.

FOOTNOTES

- **Authors' contributions:** state how each author was involved in writing the protocol.

BS conceived the study and designed the protocol with YZ, RK, DC, TK, DAW and DA. YZ communicated with the Regulatory Authorities, initiated the study and wrote the first draft of the manuscript. All authors reviewed and approved this manuscript for submission.

- **Funding statement:**

This project is supported by Versus Arthritis (Grant reference number: 20777).

- **Competing interests statement:**

None declared.

REFERENCES

1. Hunter DJ, Bierma-Zeinstra S. Osteoarthritis. *The Lancet* 2019;393(10182):1745-59. doi: https://doi.org/10.1016/S0140-6736(19)30417-9
2. Quicke JG, Conaghan PG, Corp N, et al. Osteoarthritis year in review 2021: epidemiology & therapy. *Osteoarthritis and cartilage* 2022;30(2):196-206. doi: https://doi.org/10.1016/j.joca.2021.10.003
3. Jackson J, Iyer R, Mellor J, et al. The Burden of Pain Associated with Osteoarthritis in the Hip or Knee from the Patient's Perspective: A Multinational Cross-Sectional

For peer review only

Study. *Advances in therapy* 2020;37(9):3985-99. doi: 10.1007/s12325-020-01445-4 [published Online First: 2020/07/31]

4. Makarczyk MJ, Gao Q, He Y, et al. Current Models for Development of Disease-Modifying Osteoarthritis Drugs. *Tissue engineering Part C, Methods* 2021;27(2):124-38. doi: 10.1089/ten.TEC.2020.0309 [published Online First: 2021/01/07]

5. Wylde V, Beswick A, Bruce J, et al. Chronic pain after total knee arthroplasty. *EFORT open reviews* 2018;3(8):461-70. doi: 10.1302/2058-5241.3.180004

6. Finan PH, Buenaver LF, Bounds SC, et al. Discordance between pain and radiographic severity in knee osteoarthritis: findings from quantitative sensory testing of central sensitization. *Arthritis and rheumatism* 2013;65(2):363-72. doi: 10.1002/art.34646

7. Arendt-Nielsen L. Pain sensitisation in osteoarthritis. *Clinical and experimental rheumatology* 2017;35 Suppl 107(5):68-74. [published Online First: 2017/10/03]

8. Arendt-Nielsen L, Skou ST, Nielsen TA, et al. Altered Central Sensitization and Pain Modulation in the CNS in Chronic Joint Pain. *Current osteoporosis reports* 2015;13(4):225-34. doi: 10.1007/s11914-015-0276-x [published Online First: 2015/06/01]

For peer review only

9. Lluch E, Torres R, Nijs J, et al. Evidence for central sensitization in patients with osteoarthritis pain: A systematic literature review. *European Journal of Pain* 2014;18(10):1367-75. doi: 10.1002/j.1532-2149.2014.499.x

10. Skou ST, Graven-Nielsen T Fau - Rasmussen S, Rasmussen S Fau - Simonsen OH, et al. Facilitation of pain sensitization in knee osteoarthritis and persistent post-operative pain: a cross-sectional study. (1532-2149 (Electronic))

11. Kurien T, Arendt-Nielsen L, Petersen KK, et al. Preoperative Neuropathic Pain-like Symptoms and Central Pain Mechanisms in Knee Osteoarthritis Predicts Poor Outcome 6 Months After Total Knee Replacement Surgery. *The Journal of Pain* 2018;19(11):1329-41. doi: https://doi.org/10.1016/j.jpain.2018.05.011

12. Creamer P, Hunt M, Dieppe P. Pain mechanisms in osteoarthritis of the knee: effect of intraarticular anesthetic. *The Journal of rheumatology* 1996;23(6):1031-6. [published Online First: 1996/06/01]

13. Hassan BS. Effect of pain reduction on postural sway, proprioception, and quadriceps strength in subjects with knee osteoarthritis. *Annals of the Rheumatic Diseases* 2002;61(5):422-28. doi: 10.1136/ard.61.5.422

For peer review only

14. Andreae MH, Andreae DA. Regional anaesthesia to prevent chronic pain after surgery: a Cochrane systematic review and meta-analysis. *British journal of anaesthesia* 2013;111(5):711-20. doi: 10.1093/bja/aet213 [published Online First: 2013/07/03]
15. Eldridge SM, Chan CL, Campbell MJ, et al. CONSORT 2010 statement: extension to randomised pilot and feasibility trials. *BMJ* 2016;355:i5239. doi: 10.1136/bmj.i5239
16. Chan AW, Tetzlaff JM, Altman DG, et al. SPIRIT 2013 statement: defining standard protocol items for clinical trials. *Ann Intern Med* 2013;158(3):200-7. doi: 10.7326/0003-4819-158-3-201302050-00583 [published Online First: 2013/01/09]
17. Sim J, Lewis M. The size of a pilot study for a clinical trial should be calculated in relation to considerations of precision and efficiency. *Journal of clinical epidemiology* 2012;65(3):301-8. doi: 10.1016/j.jclinepi.2011.07.011 [published Online First: 2011/12/16]
18. Billingham SA, Whitehead Al Fau - Julious SA, Julious SA. An audit of sample sizes for pilot and feasibility trials being undertaken in the United Kingdom registered in the United Kingdom Clinical Research Network database. (1471-2288 (Electronic))

For peer review only

19. Whitehead AL, Julious SA, Cooper CL, et al. Estimating the sample size for a pilot randomised trial to minimise the overall trial sample size for the external pilot and main trial for a continuous outcome variable. (1477-0334 (Electronic))

20. Hoffmann TC, Glasziou PP, Boutron I, et al. Better reporting of interventions: template for intervention description and replication (TIDieR) checklist and guide. *BMJ : British Medical Journal* 2014;348:g1687. doi: 10.1136/bmj.g1687

21. Katz JA, Kaeding CS, Hill JR, et al. The pharmacokinetics of bupivacaine when injected intra-articularly after knee arthroscopy. *Anesthesia and analgesia* 1988;67(9):872-5. [published Online First: 1988/09/01]

22. Freynhagen R, Baron R, Gockel U, et al. painDETECT: a new screening questionnaire to identify neuropathic components in patients with back pain. *Current medical research and opinion* 2006;22(10):1911-20. doi: 10.1185/030079906x132488 [published Online First: 2006/10/07]

23. Sullivan MJL, Bishop SR, Pivik J. The Pain Catastrophizing Scale: Development and validation. *Psychological Assessment* 1995;7(4):524-32. doi: 10.1037/1040-3590.7.4.524

24. Akin-Akinyosoye K, James RJE, McWilliams DF, et al. The Central Aspects of Pain in the Knee (CAP-Knee)

For peer review only

questionnaire; a mixed-methods study of a self-report instrument for assessing central mechanisms in people with knee pain. (1522-9653 (Electronic))

25. Beck At Fau - Ward CH, Ward Ch Fau - Mendelson M, Mendelson M Fau - Mock J, et al. An inventory for measuring depression. (0003-990X (Print))

26. Hawker GA, Davis AM, French MR, et al. Development and preliminary psychometric testing of a new OA pain measure – an OARSI/OMERACT initiative. *Osteoarthritis and cartilage* 2008;16(4):409-14. doi: https://doi.org/10.1016/j.joca.2007.12.015

27. Herdman M, Gudex C, Lloyd A, et al. Development and preliminary testing of the new five-level version of EQ-5D (EQ-5D-5L). *Quality of Life Research* 2011;20(10):1727-36. doi: 10.1007/s11136-011-9903-x

28. Dawson J, Fitzpatrick R, Murray D, et al. Questionnaire on the perceptions of patients about total knee replacement. *The Journal of Bone and Joint Surgery British volume* 1998;80-B(1):63-69. doi: 10.1302/0301-620X.80B1.0800063

29. Smyth C. The Pittsburgh Sleep Quality Index (PSQI). (0098-9134 (Print))

30. Rathleff MS, Petersen KK, Arendt-Nielsen L, et al. Impaired Conditioned Pain Modulation in Young Female Adults with Long-Standing Patellofemoral Pain: A Single Blinded

For peer review only

Cross-Sectional Study. *Pain medicine (Malden, Mass)* 2016;17(5):980-8. doi: 10.1093/pm/pnv017 [published Online First: 2016/01/28]

31. Vaegter HB, Graven-Nielsen T. Pain modulatory phenotypes differentiate subgroups with different clinical and experimental pain sensitivity. *Pain* 2016;157(7):1480-8. doi: 10.1097/j.pain.0000000000000543 [published Online First: 2016/03/11]

32. Petersen KK, Arendt-Nielsen L, Finocchietti S, et al. Age Interactions on Pain Sensitization in Patients With Severe Knee Osteoarthritis and Controls. *The Clinical journal of pain* 2017;33(12):1081-87. doi: 10.1097/AJP.0000000000000495 [published Online First: 2017/03/10]

33. Hunter DJ, Guermazi A, Lo GH, et al. Evolution of semi-quantitative whole joint assessment of knee OA: MOAKS (MRI Osteoarthritis Knee Score). *Osteoarthritis and cartilage* 2011;19(8):990-1002. doi: 10.1016/j.joca.2011.05.004 [published Online First: 2011/06/08]

34. Cottam WJ, Iwabuchi SJ, Drabek MM, et al. Altered connectivity of the right anterior insula drives the pain connectome changes in chronic knee osteoarthritis. *Pain* 2018;159(5):929-38. doi:

For peer review only

10.1097/j.pain.0000000000001209 [published Online First: 2018/03/21]

35. Iwabuchi SJ, Xing Y, Cottam WJ, et al. Brain perfusion patterns are altered in chronic knee pain: a spatial covariance analysis of arterial spin labelling MRI. *Pain* 2020 doi: 10.1097/j.pain.0000000000001829 [published Online First: 2020/02/19]

36. Drabek MM, Cottam WJ, Iwabuchi SJ, et al. Prescription opioid-related alterations to amygdalar and thalamic functional networks in chronic knee pain: A retrospective case control resting-state connectivity study. *medRxiv* 2022:2022.03.01.22271607. doi: 10.1101/2022.03.01.22271607

37. Bird SB, Dickson EW. Clinically significant changes in pain along the visual analog scale. *Annals of Emergency Medicine* 2001;38(6):639-43. doi: 10.1067/mem.2001.118012

38. NUH_NHS_Trust. Adverse Event Reporting SOP 2022 [Available from: https://www.nuh.nhs.uk/download/doc/docm93jijm4n3393.pdf?ver=27107 accessed 01 October 2022.

For peer review only

Figure legends:

Figure 1: Overall trial flow

Key: TKR - total knee replacement, OA - osteoarthritis, PIS - participant information sheet, SPMIC - Sir Peter Mansfield Imaging Centre, QMC - Queen's Medical Centre, QST - quantitative sensory testing, fMRI - functional magnetic resonance imaging.

For peer review only

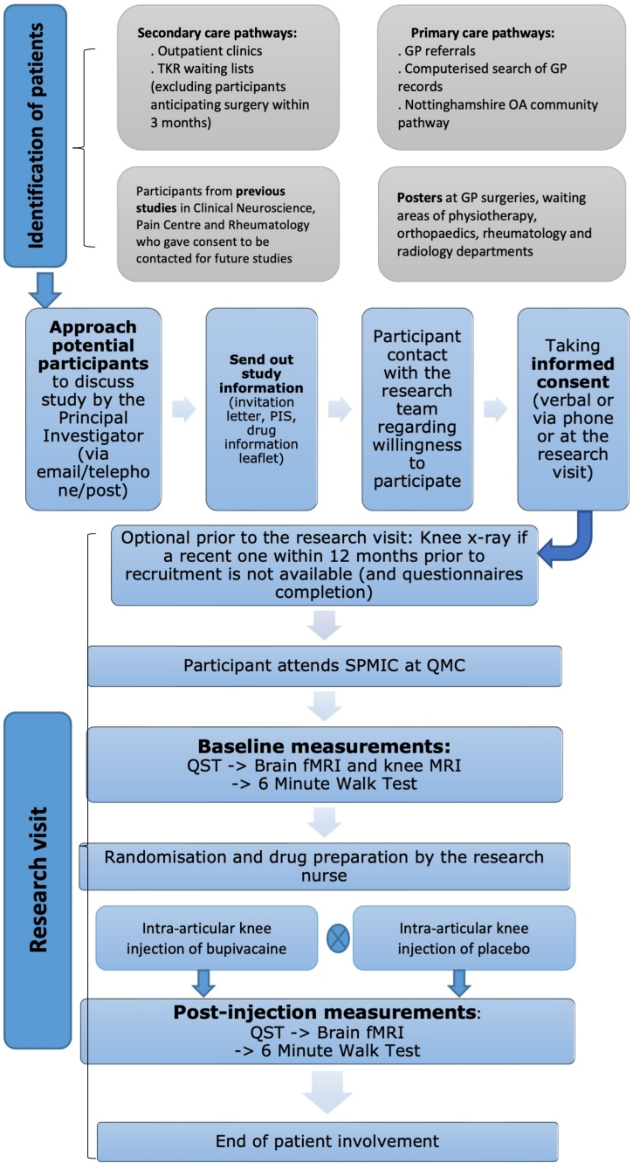

Figure 1: Overall trial flow
Key: TKR - total knee replacement, OA - osteoarthritis, PIS - participant information sheet, SPMIC - Sir Peter Mansfield Imaging Centre, QMC - Queen's Medical Centre, QST - quantitative sensory testing, fMRI - functional magnetic resonance imaging.

175x230mm (330 x 330 DPI)

SPIRIT

STANDARD PROTOCOL ITEMS: RECOMMENDATIONS FOR INTERVENTIONAL TRIALS

SPIRIT 2013 Checklist: Recommended items to address in a clinical trial protocol and related documents*

Section/item	Item No	Description	Page and Line Number
**Administrative information**			
Title	1	Descriptive title identifying the study design, population, interventions, and, if applicable, trial acronym	Page 1: Line 1-4
Trial registration	2a	Trial identifier and registry name. If not yet registered, name of intended registry	Page 2: Line 38
	2b	All items from the World Health Organization Trial Registration Data Set	N/A
Protocol version	3	Date and version identifier	Page 6: Line 193
Funding	4	Sources and types of financial, material, and other support	Page 10: Line 370
Roles and responsibilities	5a	Names, affiliations, and roles of protocol contributors	Page 10: Line 365-367
	5b	Name and contact information for the trial sponsor	Page 9: Line 301
	5c	Role of study sponsor and funders, if any, in study design; collection, management, analysis, and interpretation of data; writing of the report; and the decision to submit the report for publication, including whether they will have ultimate authority over any of these activities	Page 9: Line 300-303<br>Page 10: Line 336-339, Line 341-348
	5d	Composition, roles, and responsibilities of the coordinating centre, steering committee, endpoint adjudication committee, data management team, and other individuals or groups overseeing the trial, if applicable (see Item 21a for data monitoring committee)	Page 9: Line 297-303
**Introduction**			

For peer review only

Background and rationale	6a	Description of research question and justification for undertaking the trial, including summary of relevant studies (published and unpublished) examining benefits and harms for each intervention	Page 3
	6b	Explanation for choice of comparators	Page 3: Line 81-85
Objectives	7	Specific objectives or hypotheses	Page 4: Line 90-112
Trial design	8	Description of trial design including type of trial (eg, parallel group, crossover, factorial, single group), allocation ratio, and framework (eg, superiority, equivalence, noninferiority, exploratory)	page 4: Line 115-120

Methods: Participants, interventions, and outcomes

Study setting	9	Description of study settings (eg, community clinic, academic hospital) and list of countries where data will be collected. Reference to where list of study sites can be obtained	Page 4: Line 122-124
Eligibility criteria	10	Inclusion and exclusion criteria for participants. If applicable, eligibility criteria for study centres and individuals who will perform the interventions (eg, surgeons, psychotherapists)	Page 5: Line 133-149
Interventions	11a	Interventions for each group with sufficient detail to allow replication, including how and when they will be administered	Page 6-8: Line 188-261
	11b	Criteria for discontinuing or modifying allocated interventions for a given trial participant (eg, drug dose change in response to harms, participant request, or improving/worsening disease)	Page 5: Line 164-166
	11c	Strategies to improve adherence to intervention protocols, and any procedures for monitoring adherence (eg, drug tablet return, laboratory tests)	Page 8: Line 282-292
	11d	Relevant concomitant care and interventions that are permitted or prohibited during the trial	page 6: Line 207

For peer review only - http://bmjopen.bmj.com/site/about/guidelines.xhtml

Outcomes	12	Primary, secondary, and other outcomes, including the specific measurement variable (eg, systolic blood pressure), analysis metric (eg, change from baseline, final value, time to event), method of aggregation (eg, median, proportion), and time point for each outcome. Explanation of the clinical relevance of chosen efficacy and harm outcomes is strongly recommended	Page 8: Line 263-292
Participant timeline	13	Time schedule of enrolment, interventions (including any run-ins and washouts), assessments, and visits for participants. A schematic diagram is highly recommended (see Figure)	Page 5: Figure 1
Sample size	14	Estimated number of participants needed to achieve study objectives and how it was determined, including clinical and statistical assumptions supporting any sample size calculations	Page 4-5: Line 126-131
Recruitment	15	Strategies for achieving adequate participant enrolment to reach target sample size	Page 5: Line 151-159

Methods: Assignment of interventions (for controlled trials)

Allocation:

Sequence generation	16a	Method of generating the allocation sequence (eg, computer-generated random numbers), and list of any factors for stratification. To reduce predictability of a random sequence, details of any planned restriction (eg, blocking) should be provided in a separate document that is unavailable to those who enrol participants or assign interventions	Page 4-5: Line 168-177
Allocation concealment mechanism	16b	Mechanism of implementing the allocation sequence (eg, central telephone; sequentially numbered, opaque, sealed envelopes), describing any steps to conceal the sequence until interventions are assigned	Page 5: Line 177-180
Implementation	16c	Who will generate the allocation sequence, who will enrol participants, and who will assign participants to interventions	Page 5: Line 177-180

Blinding (masking)	17a	Who will be blinded after assignment to interventions (eg, trial participants, care providers, outcome assessors, data analysts), and how	Page 5: Line 182-186
	17b	If blinded, circumstances under which unblinding is permissible, and procedure for revealing a participant's allocated intervention during the trial	Page 6: Line 187-189
**Methods: Data collection, management, and analysis**			
Data collection methods	18a	Plans for assessment and collection of outcome, baseline, and other trial data, including any related processes to promote data quality (eg, duplicate measurements, training of assessors) and a description of study instruments (eg, questionnaires, laboratory tests) along with their reliability and validity, if known. Reference to where data collection forms can be found, if not in the protocol	Page 7-8: Line 213-264
	18b	Plans to promote participant retention and complete follow-up, including list of any outcome data to be collected for participants who discontinue or deviate from intervention protocols	N/A
Data management	19	Plans for data entry, coding, security, and storage, including any related processes to promote data quality (eg, double data entry; range checks for data values). Reference to where details of data management procedures can be found, if not in the protocol	Page 9: Line 297-303
Statistical methods	20a	Statistical methods for analysing primary and secondary outcomes. Reference to where other details of the statistical analysis plan can be found, if not in the protocol	Page 9: Line 305-333
	20b	Methods for any additional analyses (eg, subgroup and adjusted analyses)	Page 9: Line 327-333
	20c	Definition of analysis population relating to protocol non-adherence (eg, as randomised analysis), and any statistical methods to handle missing data (eg, multiple imputation)	Page 9: Line 325-326
**Methods: Monitoring**			

For peer review only - http://bmjopen.bmj.com/site/about/guidelines.xhtml

Data monitoring	21a	Composition of data monitoring committee (DMC); summary of its role and reporting structure; statement of whether it is independent from the sponsor and competing interests; and reference to where further details about its charter can be found, if not in the protocol. Alternatively, an explanation of why a DMC is not needed	Data will be monitored by the Sponsor (Page 9: Line 297-303)
	21b	Description of any interim analyses and stopping guidelines, including who will have access to these interim results and make the final decision to terminate the trial	N/A
Harms	22	Plans for collecting, assessing, reporting, and managing solicited and spontaneously reported adverse events and other unintended effects of trial interventions or trial conduct	Page 10: Line 335-339
Auditing	23	Frequency and procedures for auditing trial conduct, if any, and whether the process will be independent from investigators and the sponsor	Page 9: Line 304-305
**Ethics and dissemination**			
Research ethics approval	24	Plans for seeking research ethics committee/institutional review board (REC/IRB) approval	Page 2: Line 32
Protocol amendments	25	Plans for communicating important protocol modifications (eg, changes to eligibility criteria, outcomes, analyses) to relevant parties (eg, investigators, REC/IRBs, trial participants, trial registries, journals, regulators)	Implied in page 9: Line 297-301 as the study will follow the standardised procedures and policies of the Sponsor
Consent or assent	26a	Who will obtain informed consent or assent from potential trial participants or authorised surrogates, and how (see Item 32)	Page 5: Line 161-166
	26b	Additional consent provisions for collection and use of participant data and biological specimens in ancillary studies, if applicable	N/A
Confidentiality	27	How personal information about potential and enrolled participants will be collected, shared, and maintained in order to protect confidentiality before, during, and after the trial	Page 9: Line 298-300

For peer review only

Declaration of interests	28	Financial and other competing interests for principal investigators for the overall trial and each study site	Page 11: Line 374-375
Access to data	29	Statement of who will have access to the final trial dataset, and disclosure of contractual agreements that limit such access for investigators	Page 9: Line 297-302
Ancillary and post-trial care	30	Provisions, if any, for ancillary and post-trial care, and for compensation to those who suffer harm from trial participation	N/A
Dissemination policy	31a	Plans for investigators and sponsor to communicate trial results to participants, healthcare professionals, the public, and other relevant groups (eg, via publication, reporting in results databases, or other data sharing arrangements), including any publication restrictions	Page 10: Line 353-355
	31b	Authorship eligibility guidelines and any intended use of professional writers	Page 10: Line 355-357
	31c	Plans, if any, for granting public access to the full protocol, participant-level dataset, and statistical code	N/A
**Appendices**			
Informed consent materials	32	Model consent form and other related documentation given to participants and authorised surrogates	Supplementary B
Biological specimens	33	Plans for collection, laboratory evaluation, and storage of biological specimens for genetic or molecular analysis in the current trial and for future use in ancillary studies, if applicable	N/A

*It is strongly recommended that this checklist be read in conjunction with the SPIRIT 2013 Explanation & Elaboration for important clarification on the items. Amendments to the protocol should be tracked and dated. The SPIRIT checklist is copyrighted by the SPIRIT Group under the Creative Commons "Attribution-NonCommercial-NoDerivs 3.0 Unported" license.

For peer review only

Nottingham University Hospitals NHS Trust

Participant Consent Form

Version: 1.1 – Date: 04/01/2021 – IRAS number: 270642

Understanding Pain Mechanisms in Knee OA — UP- KNEE study

Full Title: Is there a difference in the analgesic response to intra-articular bupivacaine injection in people with knee osteoarthritis pain with or without central sensitisation?: a feasibility randomised controlled trial

Chief Investigator: Professor Brigitte Scammell (Nottingham University Hospitals NHS Trust)
Principal Investigator: Dr Yasmine Zedan

Participant Study ID: …..……………….. Initials: ………………

Participant initial each box

1. I confirm that I have read and understand the information sheet dated ________ (version _____) and the additional information leaflet dated ________ (version _____) for the above study and have had the opportunity to ask questions. ☐

2. I understand that my participation is voluntary and that I am free to withdraw at any time without my medical care or legal rights being affected. ☐

3. I understand that my medical records may be looked at by authorised individuals from the Sponsor for the study, the research group and the UK Regulatory Authority in order to check that the study is being carried out correctly. ☐

4. I understand that even if I withdraw from the above study, the data collected from me will be used in analysing the results of the trial. ☐

5. I consent to the storage including electronic, of personal information for the purposes of this study. I understand that any information that could identify me will be kept strictly confidential and that no personal information will be included in the study report or other publication. ☐

6. I understand that my participation in the study will involve having an injection of either a local anaesthetic (bupivacaine) or placebo (a mixture of salt and water) into my knee joint. ☐

7. I understand that participation in the study will involve MRI scans of my brain and knee and an x-ray of my knee (if needed). ☐

8. I agree that my GP, or any other doctor treating me, will be notified of my participation in this study. ☐

9. I understand that should there be any abnormal findings on either the fMRI scans, knee MRI or x-rays taken as part of this study, I agree to my GP being contacted. ☐

10. I agree to take part in the study. ☐

11. I agree to being contacted regarding future research studies (**OPTIONAL**) YES ☐ NO ☐

______________________	__________	______________________
Name of the patient *(Print)*	date	Patient's signature
Name of person receiving consent *(Print)*	date	Signature

Original to be retained and filed in the site file. 1 copy to patient, 1 copy to be filed in patient's notes.

For peer review only - http://bmjopen.bmj.com/site/about/guidelines.xhtml

Supplementary C

Is there a difference in the analgesic response to intra-articular bupivacaine injection in people with knee osteoarthritis pain with or without central sensitisation?: a feasibility randomised controlled trial

Template for intervention description and replication (TIDieR) checklist[1]

1. **BRIEF NAME:**
   Intra-articular knee injection with bupivacaine/placebo
2. **WHY:**
   There is a need for a better mechanistic understanding of individual factors affecting pain relief, consequently informing personalised treatment guidelines. The aim of this research is to conduct a feasibility study in order to provide a strategy for a future randomised clinical trial by comparing the effect of a peripherally targeted intervention (intra-articular bupivacaine injection) versus placebo in pre-surgical knee OA with and without signs of centrally mediated pain mechanisms. We have selected intra-articular injection of bupivacaine to achieve analgesia because this was shown to reduce knee pain in participants with knee OA one hour after injection.[2,3]
3. **WHAT**:
   Materials:
   Bupivacaine is used in clinical practice (combined with corticosteroids) for pain relief. This study does not have the intention to treat. This is not a clinical trial of an IMP.
   Description of bupivacaine (active drug):
   Bupivacaine is a local anaesthetic agent with a slow onset of action of approximately 2-5 minutes after injection and its effects last longer between approximately 2-4 hours after wash out for single knee injection. Bupivacaine binds to the intracellular portion of sodium channels and blocks sodium influx into nerve cells, preventing depolarization and the generation and conduction of nerve impulses. Its chemical designation is 1-butyl-N-(2,6-dimethylphenyl) piperidine-2-carboxamide. The empirical formula is C18H28N20, which corresponds to a molecular mass of 288.43 g/mol. Its pharmaceutical form is in a white crystalline powder that is freely soluble in 95 per cent ethanol, soluble in water, and slightly soluble in chloroform or acetone.

   The substance for placebo is sodium chloride (5 ml, 9mg/ml, 0.9% solution for injection).

   Procedures: Bupivacaine/sodium chloride will be administered in a setting fully equipped for the monitoring and support of respiratory and cardiovascular function. For intra-articular injection, the dosage of bupivacaine (5 ml, 0.25%w/v bupivacaine) and sodium chloride (5 ml, 0.9% solution for injection) will be the same for each participant.
4. **WHO PROVIDED:**
   Bupivacaine/sodium chloride will be administered by personnel experienced in their use, with training from the Nottingham University Hospitals NHS Trust, and have also been trained in advanced life support and anaphylaxis management.

For peer review only

5. **HOW:**
   Bupivacaine/sodium chloride will be administered by the intra-articular route.
6. **WHERE:**
   Bupivacaine/sodium chloride will be administered in a setting fully equipped for the monitoring and support of respiratory and cardiovascular function under totally aseptic conditions.
7. **WHEN and HOW MUCH:**
   The study involves a single visit which will include an assessment to check patients' suitability to undergo intervention with bupivacaine/placebo intra-articular knee injection. During the visit, all participants will be invited to undertake the following assessments: 1) a suite of psychometric questionnaires; 2) Quantitative Sensory Testing; 3) MRI scan of the knee and brain; 4) a 6-Minute Walk Test; and 5) a single intra-articular injection of either bupivacaine (5 ml, 0.25%w/v bupivacaine) or sodium chloride (5 ml, 0.9% solution for injection) into the index knee. Assessments will be repeated post intra-articular injection apart from the knee MRI scan.
8. **TAILORING:**
   N/A
9. **HOW WELL:**
   Planned:
   All clinicians who will deliver the intra-articular injection in this study have been trained in Nottingham University Hospitals NHS Trust, and have also been trained in advanced life support and anaphylaxis management. The clinicians will adhere to a standardised protocol of injection. Protocol completion rates will be collected as one of the study outcomes.

References

1. Hoffmann TC, Glasziou PP, Boutron I, et al. Better reporting of interventions: template for intervention description and replication (TIDieR) checklist and guide. *BMJ*. 2014;348(mar07 3):g1687-g1687. doi:10.1136/bmj.g1687
2. Hassan BS, Doherty SA, Mockett S, Doherty M. Effect of pain reduction on postural sway, proprioception, and quadriceps strength in subjects with knee osteoarthritis. *Ann Rheum Dis*. 2002;61(5):422-428. doi:10.1136/ard.61.5.422
3. Creamer P, Hunt M, Dieppe P. Pain mechanisms in osteoarthritis of the knee: effect of intraarticular anesthetic. *J Rheumatol*. 1996;23(6):1031-1036.

For peer review only

Supplementary D:

Quantitative Sensory Testing (QST) protocol:
This protocol follows standardised methodology described in previous studies.[1–5]

Pressure Algometry
To examine pressure pain thresholds (PPT), a handheld pressure algometer (Somedic AB, Sösdala, Sweden) will be used. Pressure will be increased by 30kPa/s until the participant perceives a change of the stimulus from a pressure sensation to pain sensation. The participant will then press a response button and the algometer probe will be immediately withdrawn. The PPT score in kPa is the pressure applied at the time the response button was pressed.
PPT will be recorded from the following sites:
1. Medial joint line of the index knee (the most painful knee): 2 cm proximal to superomedial patellar margin.
2. Lateral joint line of the index knee: 2 cm proximal to superolateral patellar margin.
3. The tibialis anterior muscle as a distant site to examine spreading sensitization.
2. the extensor carpi radialis longus (ECRL) muscle as a remote site to examine widespread hyperalgesia.
A practise test will be done on each site then PPTs will be recorded in triplicates which will then be averaged per site for further analysis.

Cuff Pressure Algometry
A cuff algometer (NociTech and Aalborg University, Denmark) comprised of two 13 cm single chamber tourniquet cuffs (VBM, Sulz, Germany) will use computer-controlled cuff pressure stimuli to assess pain sensitivity of the deep tissue. The device is connected to an air compressor and an electronic VAS rating system (Aalborg University) which is sampled at 10 Hz.
the cuff will be applied at the level of the ipsilateral gastrocnemius muscle and will be inflated at a rate of 1kPa/s until max pressure limit of 100 kPa. The participant will rate his pressure-induced pain severity continuously using the VAS rating system and will press a button to release the pressure. The cuff pain detection threshold (PDT) is the pressure value when the subject rated pain as 1 cm on the VAS rating system.
The cuff pain tolerance threshold (PTT) is the value of maximum pressure at the point the subject had to press the release button because of intolerable pain severity.

Temporal Summation of Pain (TSP) by Cuff Algometry
Ten consecutive cuff pressure stimulations comprised of 1-second stimulus with a 2-second inter-stimulus-interval with an intensity equal to the PTT score will be applied ipsilateral to the index knee. Participants will rate the pain severity continuously throughout the stimulation using the VAS slider and to not return the VAS slider to zero in between cuff stimulations. A constant pressure of 5 kPa is retained by the cuff in-between stimulations to ensure that fixed position of the cuff on the leg during the examination. The VAS score is recorded immediately after each individual cuff stimulus.
TSP score is calculated by subtracting the mean VAS score of the first to 4th cuff stimulations (VAS-I) from the mean VAS score of the 8th to 10th cuff stimulations (VAS-II).

For peer review only

Conditioning Pain Modulation by Cuff Algometry

A painful conditioning stimulus will be applied contralateral to the index knee using the cuff with an inflation pressure equals 70% of the participant's cuff PTT. As a test stimulus, PDT will be simultaneously examined on the ipsilateral side using the second cuff. Conditioning pain modulation (CPM) will be calculated by subtracting the unconditioned PDT score from the conditioned PDT score.

References

1. Petersen KK, Arendt-Nielsen L, Finocchietti S, et al. Age Interactions on Pain Sensitization in Patients With Severe Knee Osteoarthritis and Controls. *Clin J Pain*. 2017;33(12):1081-1087. doi:10.1097/AJP.0000000000000495
2. Rathleff MS, Petersen KK, Arendt-Nielsen L, Thorborg K, Graven-Nielsen T. Impaired Conditioned Pain Modulation in Young Female Adults with Long-Standing Patellofemoral Pain: A Single Blinded Cross-Sectional Study. *Pain Med*. 2016;17(5):980-988. doi:10.1093/pm/pnv017
3. Vaegter HB, Graven-Nielsen T. Pain modulatory phenotypes differentiate subgroups with different clinical and experimental pain sensitivity. *Pain*. 2016;157(7):1480-1488. doi:10.1097/j.pain.0000000000000543
4. Kurien T, Arendt-Nielsen L, Petersen KK, Graven-Nielsen T, Scammell BE. Preoperative Neuropathic Pain-like Symptoms and Central Pain Mechanisms in Knee Osteoarthritis Predicts Poor Outcome 6 Months After Total Knee Replacement Surgery. *J Pain*. 2018;19(11):1329-1341. doi:https://doi.org/10.1016/j.jpain.2018.05.011
5. Iwabuchi SJ, Xing Y, Cottam WJ, et al. Brain perfusion patterns are altered in chronic knee pain: a spatial covariance analysis of arterial spin labelling MRI. *Pain*. 2020;161(6):1255-1263. doi:10.1097/j.pain.0000000000001829

For peer review only
